# Cdc2-like kinases: structure, biological function and therapeutic targets for diseases

**DOI:** 10.1038/s41392-023-01409-4

**Published:** 2023-04-07

**Authors:** Mengqiu Song, Luping Pang, Mengmeng Zhang, Yingzi Qu, Kyle Vaughn Laster, Zigang Dong

**Affiliations:** 1 Department of Pathophysiology, School of Basic Medical Sciences, College of Medicine, Zhengzhou University, Zhengzhou, Henan 450001 China; 2 China-US (Henan) Hormel Cancer Institute, No.127, Dongming Road, Jinshui District, Zhengzhou, Henan 450008 China; 3 State Key Laboratory of Esophageal Cancer Prevention and Treatment, Zhengzhou University, Zhengzhou, Henan China; 4 Academy of Medical Sciences, College of Medicine, Zhengzhou University, Zhengzhou, Henan 450001 China; 5 Research Center of Basic Medicine, Academy of Medical Sciences, College of Medicine, Zhengzhou University, Zhengzhou, Henan 450001 China

**Keywords:** Drug development, Oncogenes

## Abstract

The CLKs (Cdc2-like kinases) belong to the dual-specificity protein kinase family and play crucial roles in regulating transcript splicing *via* the phosphorylation of SR proteins (SRSF1–12), catalyzing spliceosome molecular machinery, and modulating the activities or expression of non-splicing proteins. The dysregulation of these processes is linked with various diseases, including neurodegenerative diseases, Duchenne muscular dystrophy, inflammatory diseases, viral replication, and cancer. Thus, CLKs have been considered as potential therapeutic targets, and significant efforts have been exerted to discover potent CLKs inhibitors. In particular, clinical trials aiming to assess the activities of the small molecules Lorecivivint on knee Osteoarthritis patients, and Cirtuvivint and Silmitasertib in different advanced tumors have been investigated for therapeutic usage. In this review, we comprehensively documented the structure and biological functions of CLKs in various human diseases and summarized the significance of related inhibitors in therapeutics. Our discussion highlights the most recent CLKs research, paving the way for the clinical treatment of various human diseases.

## Introduction of CLKs

**Fig. 1:**
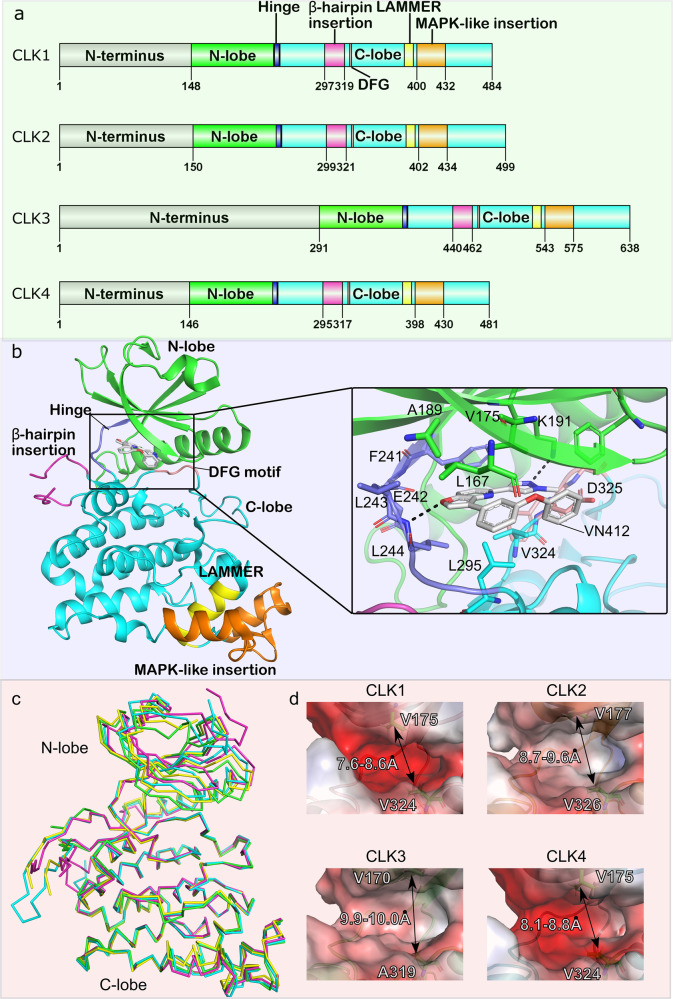
Structural comparison of human CLKs. **a** Domain structures of human CLK1, -2, -3, and -4. **b** Crystal structure of human CLK1 without N-terminus domain (PDB ID: 6I5H). The protein structure backbone is visualized as a cartoon representation, while the ligand and its interacting residues are visualized as sticks. H-bonds are shown as black dashed lines. The coloring of all domains and signature sequence motifs are consistent with panel **a**. **c** Structural superposition of CLK1 (PDB ID: 6I5H, green), CLK2 (PDB ID: 6KHE, cyan), CLK3 (PDB ID: 2EU9, magenta) and CLK4 (PDB ID: 6FYV, yellow). The protein structural backbone is represented as a ribbon. **d** Electrostatic surface representation of CLKs. The blue and red colors represent positive and negative charges, respectively. The different pockets among these kinases were determined by the distances between side chains of conserved Val residue in the N-lobe and DFG-1 residue in the C-lobe

The Cdc2-like kinases (CLKs) belong to the dual-specificity protein kinase (DSK)family, which catalyze the phosphorylation of serine, threonine, and tyrosine of substrates.^1^ The CLKs family consists of four homologous proteins (CLK1 - 4) and all family members are evolutionarily conserved in eukaryots.^2,3^ Specifically, CLKs are classified as a CMGC kinase (cyclin-dependent kinases (CDKs), mitogen-activated protein kinases (MAPKs), glycogen synthase kinases (GSKs), and CDK-like kinases) group that share the same ATP co-factor and catalyze the phosphorylation of downstream protein substrates.^4–6^ The CLK kinase domain contains an “EHLAMMERILG” motif and is located at the C-terminus of each family member. Thus, CLK proteins are also known as the “LAMMER” family^7^ (Fig. 1a). CLKs are located in the cytoplasm and nucleus, but mainly exert functions in the nuclear compartment by phosphorylating the serine / arginine (Ser-Arg) -rich domain of splicing factors.^8–10^ Generally, phosphorylation of CLKs downstream substrates is driven by recognition of the universal consensus R-x-x-S/T sequence.^3,11^ On this basis, CLKs control pre-mRNA splicing to generate different protein isoforms, which play crucial roles in cell growth and survival.^11^ SR proteins (SRSF1–12) are well-defined substrates of CLKs that bind with pre-mRNA and related spliceosome components to facilitate spliceosome assembly.^12–14^ Activated SR proteins that have been phosphorylated by CLKs participate in alternative splicing (AS) and catalyze splicing processes.^12^ On the contrary, the functional SR proteins are subsequently dephosphorylated by phosphatases. This process is required for the export of spliced mRNA from the nucleus.^12,15,16^

Among the four family members of CLKs, CLK1 has been structurally and functionally well characterized. CLK1, also termed STY, has been identified to be auto-phosphorylated at Ser / Thr / Tyr residues and phosphorylates Ser residues of its substrates.^17,18^ The catalytic domain of CLK1 is located at the C-terminus, whereas the N-terminus contains the Ser-Arg repeats responsible for mediating the interactions with the corresponding substrates.^9^ The kinase domain of CLK1 phosphorylates not only Ser-Arg dipeptide, but also Ser-Lys and Ser-Pro sites.^19^ Functionally, CLK1 is co-localized with SR proteins in the nucleus and facilitates the conformational change of the SRSF1 protein. This process subsequently disrupts the export of SRSF1 from the nucleus to cytosol and heightens SRSF1 binding to primary transcripts.^20^ However, loss of SRSF1 phosphorylation results in decreased alternative splicing of more than 100 genes, leading to irregular gene expression.^20,21^ CLK1 abundance was shown to vary periodically over the cell cycle and reached a peak at the G2/M phase, indicating a close relationship between CLK1 expression and cell cycle progression.^21^ Similar to CLK1, CLK2, −3, and −4 were also shown to contribute to cell growth and disease occurrence via the regulation of splicing.^12^ In addition, human CLK2 was found to play a role in fatty liver disease through participating in fatty acid oxidation and ketogenesis.^22^ CLK3 was suggested to play a pivotal role in the fertilization process due to its overexpression in mature spermatozoa.^23^ CLK4 has been implicated to modulate pre-mRNA intron-retaining splicing after stress withdraw.^12,24^ Collectively, CLKs act as critical regulators and exert important functions which are essential in catalyzing splicing and modulating phosphorylation. In this review, we systematically describe the structure of CLK family members, their biological functions, and the potential roles in human diseases progression, such as neurodegenerative diseases, inflammatory diseases, viral replication, and cancer. Finally, we comprehensively summarize the related small molecular inhibitors of CLKs and their therapeutic potential in various human diseases.

## Crystal structure of CLKs

Structurally, all four members of the human CLKs share highly conserved topology. Each of the proteins is comprised of a flexible unstructured N-terminal region (around 140–300 residues) and a conserved kinase domain^3,7^ (Fig. 1a). Despite being unstructured, the N-terminal extensions of CLKs were reported to resemble similar RS (arginine / serine) domains present in their target SR protein substrates which then act as a bridge mediating the interactions between their kinase domain and the RS domain of the SR proteins. Removing this N-terminus of CLK1 results in a significant decrease of the phosphorylation level of the RS domain of SRSF1. This observation further reflects that the intrinsic disordered region of CLK1 and its mediated protein-protein contacts not only enhances the binding of the downstream proteins but also facilitates the hyperphosphorylation of the substrates.^25^

In contrast, the catalytic domain of CLKs is well-ordered and displays a typical kinase fold, containing an N-lobe and a C-lobe bridged by a hinge region forming a conserved ATP binding pocket^3,12^ (Fig. 1). The N-lobe consists of three β-strands, an α-helix, and two additional β-strands. The C-lobe possesses three conserved structural insertions defining the CLK family.^3^ These signature motifs are β-hairpin insertion, a LAMMER domain, and a mitogen-activated protein kinase (MAPK)-like insertion (Fig. 1). The extended β-hairpin insertion present at the top of the C-lobe (residues 297–319 in CLK1, 299–321 in CLK2, 440–462 in CLK3 and 295–317 in CLK4) is highly conserved in CLK family members. Another predominant sequence motif has invariant “EHLAMMERILG” region (residues 386–396 in CLK1, 388–398 in CLK2, 529–539 in CLK3 and 384–394 in CLK4), from which the family name LAMMER protein kinase is derived, located at the bottom of the C-lobe (Fig. 1). The last shared MAPK-like insertion is organized as helix-strand-strand-helix while in the same region in MAPK protein family, the two-strand β-sheet is substituted with a loop. These structural features of CLK-specific sequence motifs are likely important for their substrate recognition and binding specificity.

Structural superimposition of all human CLKs protein crystal structures demonstrated that their kinase domains share very similar topological features^3,26,27^ (Fig. 1c). Thus, CLK inhibitors are typically ATP mimetics that exert their activity through binding within the ATP pocket. As shown in Fig. 1b, the inhibitor generally contains aromatic heterocycles which are sandwiched between hydrophobic residues (L167, V175 in CLK1) of the N-lobe and hydrophobic residues (L295, V324 in CLK1) of the C-lobe resemble the binding of the adenine ring system of ATP co-factor.^28^ Chemical modifications within the ring of the inhibitors form hydrogen bonds with the main chain atom of the hinge region. The DFG sequence motif in the C-lobe was shown to be essential for inhibitor and substrate binding. It has been reported that the DFG-1 residue (Val in CLK1, 2, 4) is substituted by a shorter side chain Ala in CLK3, which leads to the increased size of the CLK3 binding cavity. This subtle difference has resulted in the development of selective inhibitors on CLK1, −2, and −4 instead of CLK3^27^ (Fig. 1d). Further electrostatic surface calculations showed distinct charge distributions in the binding pocket, indicating that this is likely another important element for selectivity which can be considered during drug design. Taken together, the subtle structural differences in the inhibitor binding site should be considered when designing selective CLK inhibitors in the future.

## Biological function of CLKs

### CLKs in regulation of the splicing process

Alternative splicing is a biological phenomenon that enables the generation of multiple mRNA and protein products from a single gene.^25^ Five main types of AS are currently known: ES (exon skipping), IR (intron retention), MXE (mutually exclusive exon), A5SS (alternative 5′ splice site), and A3SS (alternative 3′ splice site).^29,30^ The spliceosome is an enzymatic machine that is required to splice pre-mRNA.^31^ Spliceosome formation is the result of complex interactions between small nuclear ribonucleoproteins (snRNPs, including U1, U2, U4/U6, and U5) and more than 150 additional proteins.^32^ In addition, different RNA-binding proteins (RBPs) take part in alternative splicing, including SR proteins and heterogeneous nuclear ribonucleoprotein (hnRNP) family proteins.^31–35^ CLKs and SR-specific protein kinases (SRPKs) are largely responsible for phosphorylating the RS dipeptide repeat domains of the SR protein families (SRSF1–12).^36^ Once phosphorylated, SR proteins regulate RNA splicing and participate in multiple physiological functions.^37,38^ Besides, other splicing-related factors, such as RBM (RNA binding motif) proteins, are also involved in generating mature mRNA through the process of alternative splicing.^35^ Additionally, ~95% of human genes transcribe multiple mRNA isoforms by differential inclusion of exons.^39^ Here, we discuss the involvement of CLKs in regulating the splicing process through phosphorylating splicing-related proteins.

**Fig. 2:**
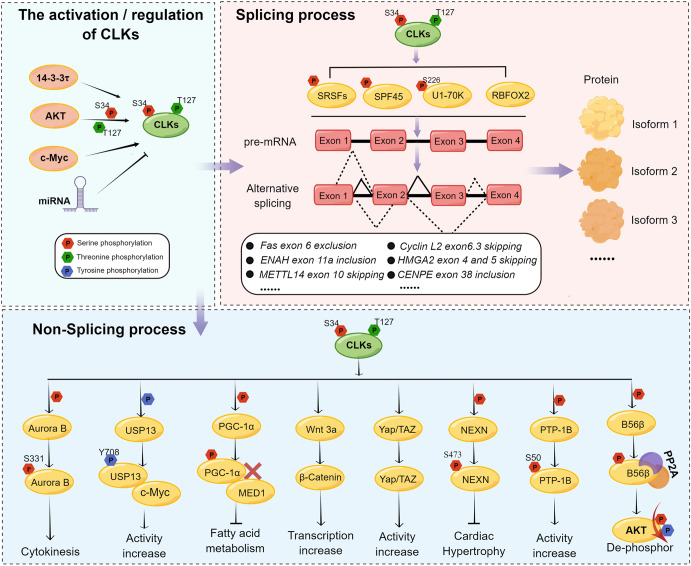
Biology function of CLKs. CLKs participate in biological processes by modulating splicing and non-splicing functions. CLKs are activated, phosphorylated or regulated by c-Myc, AKT or 14–3–3τ, or negatively regulated by miRNAs. CLKs affect their downstream effectors by phosphorylating serine, threonine, or tyrosine residues to activate cellular splicing and non-splicing processes. Alternative splicing of certain genes is increased in response to protein phosphorylation by CLKs. Targets of CLKs include SR proteins, SPF45, and/or U1–70K or modulated RBFOX2. Consequently, different protein isoforms that function in multiple cellular processes are generated. CLKs promote cytokinesis, increase c-myc activity, and suppress fatty acid metabolism by phosphorylating downstream Aurora B, USP13, and PGC-1α. The activation of Wnt/β-catenin and Hippo signaling by increased expression of Wnt 3a or YAP further highlights the importance of CLKs in non-splicing processes. The figure was generated using Figdraw (www.figdraw.com)

**Fig. 3:**
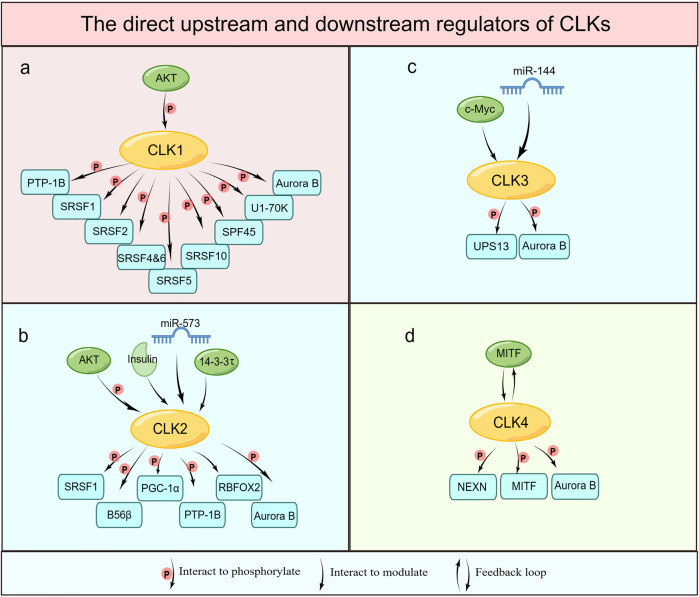
The direct upstream and downstream regulators of CLKs. The expression or activities of CLKs are directly regulated by the upstream proteins such as AKT, c-Myc, or miRNAs. CLKs phosphorylate or modulate their downstream targets, which have been demonstrated in publications, to participate in pivotal processes. The upstream and downstream regulators of CLK1 (**a**), CLK2 (**b**), CLK3 (**c**), and CLK4 (**d**) have been shown in the figure seperately. The figure was generated by Figdraw (www.figdraw.com)

Human CLK1 was shown to regulate the cellular distribution of the SR family proteins *via* the phosphorylation of their respective C-terminus (Figs. 2 and 3). Among these factors, SRSF1 is a well-characterized downstream substrate of CLKs.^25^ The N-terminus of CLK1 was shown to interact with SRSF1 through its RS domain which was necessary for the hyperphosphorylation of SRSF1. This process further facilitates the binding of SRSF1 to its RNA target Ron ESE (AGGCGGAGGAAGC), an RNA oligomer designed by the SRSF1 exonic enhancer sequence.^25^ A phosphoproteomic analysis of gastric tumor and patient-derived xenograft (PDX) samples identified that SRSF2 was hyperphosphorylated in tumor samples. Inhibition of CLK1 by inhibitors or siRNA downregulated CLK1-dependent SRSF2 splicing activity, resulting in a reduced cell proliferation, invasion, and migration of gastric cancer.^40^ The results indicate the involvement of SRSF2 in CLK1 modulating splicing signals.^40^ SRSF1 was also shown to be a substrate of CLK2, and its phosphorylation status was observed to be closely related to the expression of CLK2, which is essential for its nuclear localization and splicing functions.^41,42^ A CLK3-HMGA2 alternative splicing axis was discovered to promote the stemness potential of human hematopoietic stem cells (HSCs) in vitro and in vivo.^43^ The finding illustrated that CLK3 promoted the skipping of HMGA2-L exon in a SRSF1-dependent manner to reinforce an HSC-specific program. Mutated SRSF1 binding motif within HMGA2-L exon 4 disrupted CLK3 regulated skipping of HMGA2-L through SRSF1, providing a direct molecular link between CLK3 and SRSF1.^43^ CLK1 phosphorylated SRSF4 and SRSF6 during insulin stimulation resulting in protein kinase C βII (PKCβII) pre-mRNA alternative splicing which subsequently participated in insulin-stimulated actin rearrangements. In addition, CLK1 overexpression promoted exon 17 inclusion splicing and increased the expression of PKCβII through phosphorylation of SR proteins in response to insulin stumilization.^5^ It was reported that CLK1 phosphorylated SRSF5 on Ser 250, thereby, affecting alternative splicing of METTL14 and Cyclin L2 to promote cell metastasis and viability in pancreatic cancer.^44^ Inhibition of the CLKs enzymatic activities preferentially inhibited the phosphorylation of SRSF10 at Ser 129, 131, and 133 eliciting p53-dependent apoptosis in human colorectal cancer cells. Moreover, the interaction between CLK1 and SRSF10 was confirmed by a GST-pull-down assay.^45^

In addition, CLK kinases were shown to phosphorylate the spliceosome-associated splicing factor 45 (SPF45).^46,47^ CLK1 phosphorylated SPF45 at eight different Ser residues, facilitating cell migration and invasion in a phosphorylation-dependent manner on SKOV-3 cells. Furthermore, CLK1 enhanced SPF45-induced *Fa*s mRNA exon 6 exclusion to facilitate serum-stimulated cell migration, whereas inhibition of CLK1 promoted SPF45 degradation and inhibited malignant phenotype in a proteasome-dependent manner.^46^ CLK1 was reported to phosphorylate U1–70K at the C-terminal Ser226 residue, facilitating U1–70K release from subnuclear granules and its subsequent interaction with U1 snRNP and SRSF1 for early spliceosomal protein assembly.^48^ Furthermore, SR protein kinase 1 (SRPK1) dissociated CLK1 from U1–70K to recycle kinase catalysis.^48^ Elevated CLK2 mRNA levels were observed in luminal and HER2 + breast cancer cell lines as well as the EGFR-amplified MDA-MB-468 and EGF-dependent MCF10DCIS triple-negative breast cancer (TNBC) cell lines. CLK2 promoted the inclusion skipping at exon 11a of mesenchymal-type ENAH in luminal breast cancer cells through the modulation of RBFOX2 as opposed to SRSF1.^42^ The usage of ENAH exon 11a affected by CLK2 subsequently implied cell growth, migration, and invasion in breast cancer.^42^

### CLKs in regulation of the nonsplicing process

Besides splicing, the CLKs are involved in other biological functions *via* phosphorylation of their substrates (Figs. 2 and 3). CLK1, −2, and −4 are required for Aurora B activation *via* its phosphorylation at the Ser331 residue during late cytokinesis in normally segregating cells.^2^ Phosphorylation of protein-tyrosine phosphatase (PTP-1B) at Ser50 by CLK1 and CLK2 promoted its enzymatic activity in ^32^P-labeled in vitro kinase assay, phosphatase assays, and co-transfection experiment in HEK293 cells.^49^ CLK2 phosphorylated PGC-1α at 11 Ser residues along its SR domain, resulting in the repression of PGC-1α transcriptional activity on gluconeogenic genes and the disruption of the physical interaction of PGC-1α with MED1. This biological process was found to attenuate PPARα transcription, decrease fatty acid oxidation, promote fatty liver disease, and suppress hepatic gluconeogenesis.^22,50^

AKT kinase exhibits a complex regulatory relationship with CLK1 and CLK2.^5,50–53^ CLK1 is a preferred substrate of AKT; the AKT phosphorylation site on CLK1 is located in its N-terminal unstructured SR domain but not the kinase domain.^5,53^ Mutation of the predicted AKT phosphorylation sites of CLK1 to alanine residues (Ser36, Thr122, and Ser139 mutated to Ala) increased PCKβII level throughout the preadipocyte differentiation to mature adipocytes process in 3T3-L1 cells. Furthermore, the S36A mutation of CLK1 resulted in the reduction of SRSF1 and SRSF2 phosphorylation, the T122A mutation led to decrease of SRSF5 phosphorylation, and the S139A mutation contributed to the diminished phosphorylation of SRSF4, SRSF6, SRSF5 as well as SRSF1 and SRSF2 in 3T3-L1 preadipocytes.^53^ Consequently, the adipogenesis program was blocked prior to differentiation in cells transfected with both CLK1 single mutants and CLK1-AAA mutations.^53^ AKT was also found to stabilize CLK2 through phosphorylation of CLK2 at Ser34 and Thr127 residues to enhance cell proliferation and block cell apoptosis in response to ionizing radiation.^52^ In turn, CLK2 was found to phosphorylate the regulatory subunit B56β of phosphatase 2 A (PP2A), which was required to drive AKT-PP2A complex formation that results in AKT dephosphorylation at the Ser473 and Thr308 residues.^50,51^ Moreover, CLK2 kinase activity was induced by AKT phosphorylation at Thr343 residue of the CLK2 activation loop in response to insulin and feeding in hepatic gluconeogenesis. Once CLK2 was activated, it underwent auto-phosphorylation to stabilize itself.^50^ Altogether, these results suggested that CLK2 functions as both an up-stream regulator and down-stream substrate of AKT, which operates as a self-regulatory loop. However, this function was probably cell type-specific because no experimental evidence confirming the interaction between CLK2 and AKT was identified in human breast cancer cells.^42^ In addition, murine CLK2 is auto-phosphorylated at a highly conserved auto-phosphorylation Ser141 site that influences its subnuclear localization; the subcellular localization is suspected to impact substrate interaction properties of CLK2.^54^ Besides, proteasomal degradation of CLK2 was decreased in glioma stem cells (GSCs) through binding with 14–3–3τ protein.^55^ Moreover, AKT/ Forkhead box O3a (FOXO3a) /p27 pathway was identified to participate in GSCs growth inhibition which caused by CLK2 knocking down.^55^ Apart from this, CLK4 bound with nexilin (NEXN) and phosphorylated it at Ser437 to regulate cardiac function, subsequently reversing pathological cardiomyocyte hypertrophy.^56^

In summary, the role of CLKs in splicing or non-splicing processes is complex and diverse. The general function of CLKs requires the interaction with or phosphorylation of their downstream or upstream proteins. In this review, we summarize the crucial related proteins and potential functions of CLKs in the regulation of cell growth, metastasis, fatty acid oxidation or cardiac function maintain by affecting the activities of downstream proteins (Table 1, Figs. 2, and 3). However, further investigation of CLKs in modulating different proteins or signaling pathways is needed to comprehensively understand the roles that CLKs play in physiological and pathological processes.

**Table 1:** Regulators of CLK proteins

Name	Interaction	Subtypes	Biological function	Ref.
**Downstream regulators**				
SRSF1	Downstream of CLK1	Hyperphosphorylation	Hyperphosphorylation of SRSF1 by CLK1 furtherly facilitated the binding of SRSF1 to Ron ESE	^25^
	Downstream of CLK2	Phosphorylation	Downregulated CLK2 expression decreased the phosphorylation status of SRSF1to inhibit its splicing function	^41,42^
	Downstream of CLK3	Splicing modulation	CLK3 strongly affected HMGA2 isoforms switching in an SRSF1-dependent manner	^43^
SRSF2	Downstream of CLK1	Splicing modulation	CLK1 inhibition decreased SRSF2 expression and downregulated CLK1-dependent SRSF2 splicing activity in gastric cancer cells	^40^
SRSF4 and SRSF6	Downstream of CLK1	Phosphorylation	CLK1(Clk/Sty) phosphorylated SRSF4 and SRSF6 during insulin stimulation resulting in exon 17 inclusion and elevated expression of PKCβII	^5^
SRSF5	Downstream of CLK1	Phosphorylation at Ser250	CLK1 promoted alternative splicing of METTL14 and Cyclin L2 in pancreatic cancer	^44^
SRSF10	Downstream of CLK1	Phosphorylation at Ser129, 131 and 133	CLKs modulated the tumorigenic activity of SRSF10 in human colorectal cancer cells	^45^
SPF45	Downstream of CLK1	Phosphorylation at eight serine residues	CLK1 enhanced SPF45-induced *Fas* mRNA exon 6 exclusion Inhibition of CLK1 promoted SPF45 degradation *via* proteasome-dependent proteolysis	^46,47^
U1–70K	Downstream of CLK1	Phosphorylation at Ser226	Facilitated U1–70K released from subnuclear granules and subsequent interaction with U1 snRNP and SRSF1	^48^
RBFOX2	Downstream of CLK2	Splicing modulation	Mediated mesenchymal-type ENAH splicing at exon 11a	^42^
PTP-1B	Downstream of CLK1/2	Phosphorylation at Ser50	Activated PTP-1B and modulated the enzymatic activity	^49^
B56β	Downstream of CLK2	Phosphorylation	Phosphorylation of B56β by CLK2 leads to AKT dephosphorylation at Ser473 and Thr308 sites	^50,51^
PGC-1α	Downstream of CLK2	Phosphorylation at 11 serine residues on SR domain	Disrupted the PGC-1α–MED1 interaction and reduced MED1 recruitment to PPARα/PGC-1α promoter resulting in decreased fatty acid oxidation CLK2 phosphorylated the SR domain of PGC-1α to repress PGC-1α transcriptional activity on gluconeogenic genes in hepatic gluconeogenesis	^49,50^ ^22,50^
USP13	Downstream of CLK3	Phosphorylation at Y708	Promoted USP13 binding with c-Myc, resulting in the activation of purine metabolic gene transcription in CCA	^124^
NEXN	Downstream of CLK4	Phosphorylation at Ser437	CLK4 phosphorylated NEXN at Ser437 to participate in cardiac function regulation	^56^
MITF	Downstream of CLK4	Phosphorylation at Tyr360	CLK4 phosphorylated MITF at Tyr360 to promote the degradation of MITF	^126^
Aurora B	Downstream of CLKs	Phosphorylation at Ser331	Activated Aurora B during late cytokinesis in normally segregating cells	^2^
**Upstream regulators**				
AKT	Upstream of CLK1	Phosphorylation CLK1 at SR domain (Ser36, Thr122 and Ser139)	AKT phosphorylated the SR domain of CLK1	^53^
	Upstream of CLK2	Phosphorylation CLK2 at Ser34, Thr127 and Thr343	AKT bond and phosphorylated CLK2 at Ser 34 and Thr 127 in response to ionizing radiation AKT phosphorylated CLK2 at Thr343 in response to insulin stimulation in hepatic gluconeogenesis	^50–52^
Insulin stimuli	Upstream of CLK2	Phosphorylation CLK2 at Ser342 and Thr343	CLK2 kinase activity was induced by insulin at Ser342/Thr343 in hepatic gluconeogenesis	^50^
miR-573	Upstream of CLK2	Regulator	miR-573 bond directly with CLK2 and significantly inhibited the expression of CLK2	^121^
14–3–3τ	Upstream of CLK2	Regulator	14–3–3τ bond with CLK2 to prevent the proteasomal degradation of CLK2 and increase protein stabilization in glioma stem cells	^55^
c-Myc	Upstream of CLK3	Transcriptional activator	c-Myc overexpression enhanced CLK3 expression	^124^
miR-144	Upstream of CLK3	Regulator	miR-144 post-transcriptionally regulated CLK3 to suppress Wnt/β-catenin signaling in hepatocellular carcinoma	^125^
MITF	Upstream of CLK4	Regulator (Feedback loop)	MITF bond to the E-boxes in the CLK4 promoter to reduce CLK4 transcript expression in ESCC	^126^

## CLKs and human diseases

**Fig. 4:**
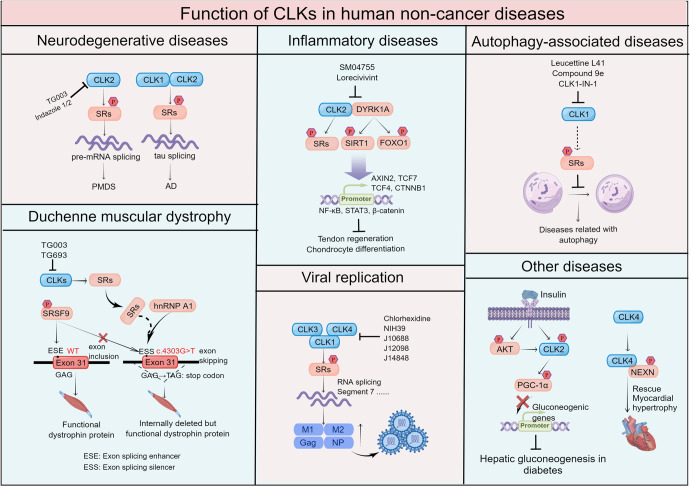
Function of CLKs and related inhibitors in human non-cancer diseases. CLKs participate in various human non-cancer diseases including neurodegenerative diseases, inflammatory diseases, Duchenne muscular dystrophy, viral replication, autophagy-associated diseases, and other diseases. CLKs mediate the pathological processes through modulating alternative splicing or changing transcriptional activities. The figure was generated by Figdraw (www.figdraw.com)

**Fig. 5:**
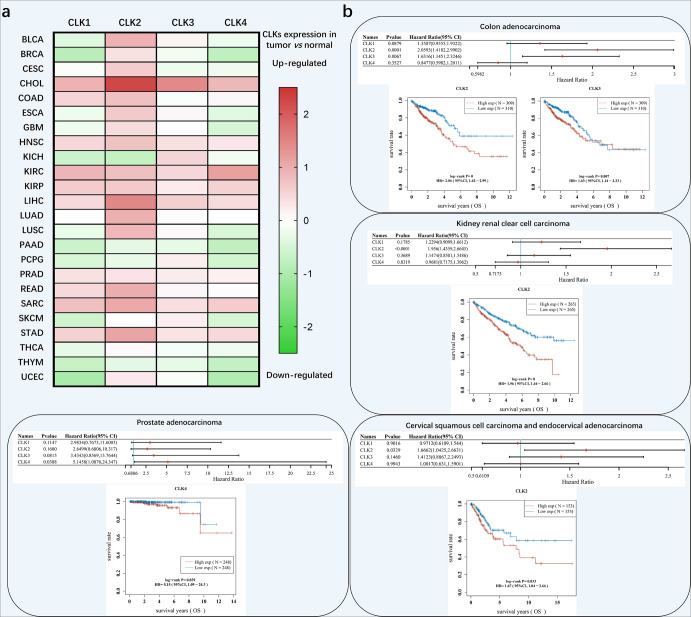
Expression status and clinical outcomes associated with CLKs transcript levels in different tumor types. **a** RNA-seq data of CLKs expression in tumor and normal tissues were obtained from the TCGA database (https://portal.gdc.cancer.gov/). The Kruskal-Wallis test was used to assess statistical significance. **b** Raw counts of clinical survival information of CLKs were obtained from The TCGA dataset (https://portal.gdc.cancer.gov/). The KM survival analysis with log-rank test was also used to compare the survival differences between the above two groups. For Kaplan–Meier curves, *p* values, and hazard ratio (HR) with 95% confidence interval (CI) were generated by log-rank tests and univariate Cox proportional hazards regression. *, ** and *** represented *p* < 0.05, *p* < 0.01 and *p* < 0.001, separately. *p* < 0.05 was considered statistically significant

**Fig. 6:**
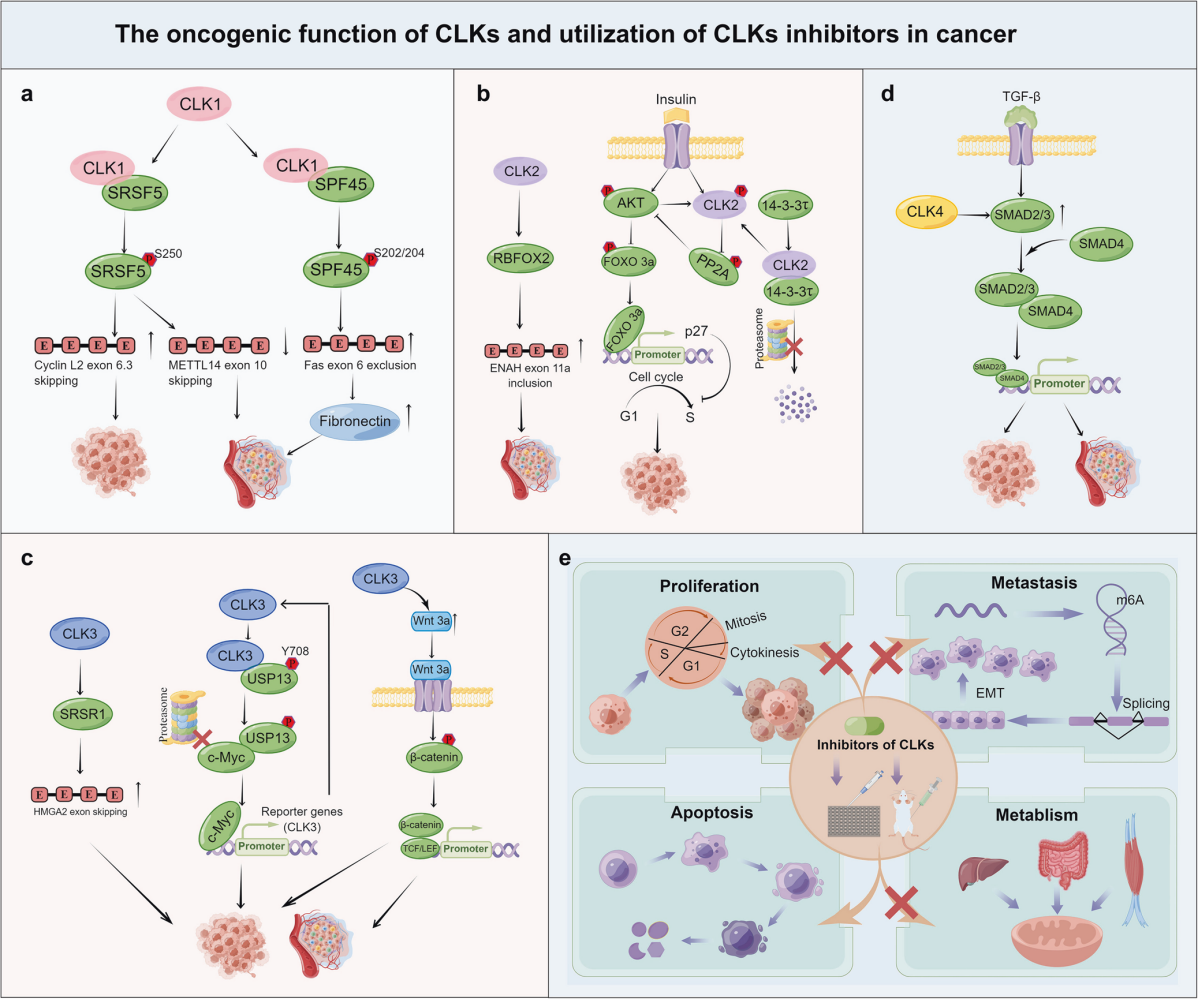
The oncogenic function of CLKs and utilization of CLKs inhibitors in cancer. **a–d** CLKs participate in cancer development, invasion, and metastasis by altering mRNA splicing, wnt/β-catenin signaling, TGF-β signaling, or mediating cell cycle transition. CLKs regulate cancer growth or metastasis by phosphorylating or modulating their downstream regulators, for instance, SRSF5, SPF45, USP13 SMAD3, PP2A, and other proteins. CLKs inhibitors decreased cancer growth, metastasis, metabolism, and promoted apoptosis by modulating genes participating in cell cycle, EMT, metabolic pathway, and apoptosis, respectively. **e** Preclinical cancer research conducted to assess the therapeutic potential of CLKs-targeting compounds showed significant tumor growth inhibitory effects. The figure was generated by Figdraw(www.figdraw.com)

The CLKs exert important functions and participate in various human noncancer diseases and cancer development through splicing or non-splicing processes.^57,58^ Here, we documented the functions of CLKs and addressed the therapeutic potential of targeting CLKs in various human diseases (Tables 2–4 and Figs. 4–6).

**Table 2:** Summary of CLKs and associated therapeutic strategies in human non-cancer diseases

Human diseases	Compounds	Targets	Phenotype	Mechanism	Cellular model	Animal model	Ref.
**Neurodegenerative diseases**							
Alzheimer’s disease (AD)	\	Human and murine CLK2	The expression and activity of CLK2 led to the misregulation of tau exon 10 splicing which contributed to AD	CLK2 related alternative splicing of tau exon 10 was regulated by phosphorylating SR proteins	Transfection of CLK2 plasmids at 2 μg significantly increased the skipping of exon 10 of tau in HEK293 cells	\	^59–62^
Phelan-McDermid syndrome (PMDS)	TG003	CLK2	CLK2 targeting improved autism and neuronal functions in PMDS	TG003 recovered spine density in *Shank3*-deficient neurons by targeting CLK2	10 μM TG003 rescued spine density in *Shank3* deficient neurons	\	^64^
	Indazole 1 and Indazole 2	CLK2		Indazole 1 and -2 elevated spine density in *Shank3*-deficient neurons by targeting CLK2	Indazole 1 and -2 significantly improved spine density at 300 nM and 1 µM.	\	^63^
**Duchenne muscular dystrophy**							
Duchenne muscular dystrophy (DMD)	TG003	CLKs and SRp30c/SRSF9	TG003 promoted exon skipping only in the c.4303 G > T mutant cells to generate partially functional dystrophin protein	Dephosphorylation of SR protein(s) cooperated with hnRNP A1 to act as a co-inhibitor during the recognition of exon 31, resulting in functional dystrophin protein	Up to 50 μM of TG003 promoted exon 31 skipping in mutated Hela cells and showed no cytotoxicity to cells	Up to 100 mg/kg per day per os showed no toxicity to mice	^68^
	TG693	CLK1	TG693 promoted the skipping of exon 31 and restored dystrophin expression in patient cells harboring the c.4303 G > T mutation	TG693 targeted CLK1 to reduce downstream SR proteins phosphorylation, resulting in exon-skipping therapy in DMD	TG693 inhibited the phosphorylation of SRSF4 at 5 μM and SRSF6 at 20 μM by targeting CLK1. Up to 20 μM of TG693 promoted the skipping of exon 31 and upregulated dystrophin expression in patient cells harboring c.4303 G > T mutation	Oral administration of 30 mg/kg TG693 reduced SRs phosphorylation, particularly SRSF4 in mice No apparent acute toxicity in rats at up to 100 mg/kg per os	^67^
**Inflammatory diseases**							
Tendinopathy	SM04755	CLK2 and DYRK1A	SM04755 treatment or knock-down of CLK2 prevented tendon destruction and promoted tendon regeneration in tendinopathy	SM04755 led to intron retention in the mRNA of Wnt pathway genes; decreased tenocyte catabolic enzyme expression; inhibited inflammatory signaling mediators NF-κB and STAT3 by targeting CLK2 and DYRK1A	0.3 μM of SM04755 inhibited inflammatory cytokine production and protected tenocytes from the catabolic breakdown	0.3 mg/cm^2^ of SM04755 inhibited inflammation and promoted tendon healing in a collagenase-induced acute tendinopathy model in rats	^69^
Knee osteoarthritis (OA)	Lorecivivint	CLK2 and DYRK1A	Inhibition or knock-down of CLK2 induced chondrocyte differentiation and suppressed cartilage catabolic enzyme expression	Lorecivivint inhibited CLK2-mediated phosphorylation of SR splicing factors and DYRK1A-mediated phosphorylation of SIRT1 and FOXO1	Lorecivivint inhibited IL-6 and TNF-α production in IL-1β and LPS-stimulated synovial fibroblasts, and LPS-stimulated THP-1 cells in a dose-dependent manner	Intra-articular injection of lorecivivint (0.1 mg, 0.3 mg, 1 mg) decreased phospho-SRSF, phospho-SIRT1, phospho-FOXO1, and phospho-STAT3 in the ACLT + pMMx model and the MIA model of rat knee OA	^71,72^
**Viral replication**							
HIV-1 virus	Chlorhexidine	CLK3 and CLK4, slightly activity against CLK2	CLK3 and CLK4 promoted the expression of HIV-1 Gag and viral RNA abundance	Chlorhexidine significantly inhibited HIV-1 Gag synthesis and suppressed HIV-1 regulatory protein Rev accumulation to prevent virus replication	2.5 μM of chlorhexidine significant repressed HIV-1 replication did not significantly impact cell viability in PBMCs	Chlorhexidine was suggested to applicate on mucosal surfaces to establish well toleration to block HIV-1 in human	^81^
Influenza A virus (IAV)	NIH39	CLK1	Inhibition or knock-down of CLK1 reduced the replication of influenza A/WSN/33	NIH39 reduced viral NS mRNA splicing ratio and inhibited viral M1, M2, and NS1 protein levels to suppress viral replication	NIH39 showed antiviral activity with an IC_50_ of 6.6 μM and reduced viral mRNA splicing and related protein expression at 12.5 μM in A549 cells	CLK1^−/−^ mice showed a reduction of viral replication compared to wild-type C57BL/6 mice	^83^
	J12098 (Corilagin)	CLK1	J12098 exhibited anti-influenza ability by targeting CLK1	J12098 showed better docking to CLK1 and closely interacted with CLK1	J12098 exerted anti-influenza virus effect with EC50 values of 2.0 ± 2.22 μg/mL a CC50 value of 153.54 μg/mL in vitro	\	^86^
	J14848 (Pinosylvin)	CLK1	J1484 exhibited anti-influenza ability in vitro by targeting CLK1	J14848 showed better docking to CLK1 and closely interacted with CLK1	J14848 exerted an anti-influenza virus effect with EC50 values of 5.28 ± 2.45 μg/mL and a CC50 value of 18.26 μg/mL in vitro	\	^86^
	J10688 (Clypearin)	CLK1	J10688 exerted anti-influenza virus activity in vivo and in vitro by potently targeting CLK1	J10688 impaired the synthesis of viral proteins NP and M2 and downregulated the phosphorylation of splicing factors SF2/ASF and SC35	J10688 exerted an anti-influenza virus effect with EC50 values of 1.2 ± 0.28 μg/mL and a CC50 value of >200 μg/mL 3, 10, and 30 μM of J10688 remarkably decreased the NP and M2 expression and reduced the copy number of viral RNA synthesis in a dose-dependent manner	Mice intravenous treated with 30, 10, and 3 mg/kg/day J10688 showed 91.67% survival rates, whereas all mice died in the normal saline group within 9 days J10688 administration reduced lung virus titer, enhanced immunological function, and alleviated influenza-induced acute lung injury	^86,87^
**Autophagy-associated diseases**							
Autophagy-associated diseases	Leucettine L41	CLK1	Leucettine L41 triggered the accumulation of LC3 foci and autophagy by targeting CLK1	Leucettine L41 treatment induced a 150-fold increase of the exon 4-containing CLK1 mRNA to inhibit the activity of CLK1 and induce autophagy	Leucettine L41 induced LC3 foci formation and triggered autophagy in U-2 OS cells without modifying the autophagic flux	\	^100^
	Compound 9e	CLK1	Compound 9e was an efficient inducer of autophagy	Compound 9e redistributed the location of SR proteins and induced the formation of autophagosome in SKOV-3 cells by targeting CLK1	Compound 9e treatment converted LC3I to LC3II and formed autophagosomes in vitro	\	^91^
	CLK1-IN-1	CLK1	CLK1-IN-1 treatment increased autophagic flux and induced autophagy	CLK1-IN-1 treatment reduced the phosphorylation and affected the subcellular redistribution of the downstream SR proteins	CLK1-IN-1 elevated the expression level of LC3II protein as well as the ratio of LC3II to LC3I in a dose- and time- dependent manner	CLK1-IN-1 showed hepatoprotective effects on the APAP-induced acute liver injury mouse model.	^104^
**Other diseases**							
Pathological cardiac hypertrophy	\	CLK4	CLK4 deletion led to pathological cardiac hypertrophy	CLK4 regulated cardiac function through phosphorylation of NEXN at Ser437 to restore heart failure	CLK4 formed a complex with downstream NEXN and phosphorylated it at Ser437	Exogenous overexpression of the NEXN (S437E) phosphorylation-mimic mutant in *Clk4*-cKO mice via AAV9 injection reversed the pathological phenotype induced by CLK4 knockout	^56^
Hyperglycemia	\	CLK2	CLK2 was induced by insulin/AKT and acted as a suppressor of hepatic gluconeogenesis	The activated CLK2 phosphorylated PGC-1α at SR domain to repress PGC-1α transcriptional activity on gluconeogenic genes	Insulin/AKT phosphorylated CLK2 at Ser342/Thr343 to stabilize CLK2.	CLK2 was downregulated in the diabetic and obese *db/db* mice (*Lepr*^*db*^*/Lepr*^*db*^). Re-introduction of CLK2 in the livers of *db/db* mice dramatically restored the glucose phenotype	^50^
Circadian body-temperature oscillations	TG003	CLK1/4	CLK1/4 acted as molecular thermometers in response to circadian body-temperature oscillations	TG003 administration abolished the re-phosphorylation of SRSF5 and SRSF6 as well as exon inclusion and intron retention events during the shift of temperature from 42 °C to 35 °C by targeting CLKs	HEK293 cells with 35 °C TG003 treatment showed a similar temperature dependent AS as 39 °C DMSO samples	Alligator CLK4 activity decreased with the temperature increasing from 27 °C to 35 °C. Turtle CLK1 showed full activity below 26 °C but lost 90% activity above 31 °C TG003 prevented the increased intron retention at a colder temperature	^109,110^

*os* oral solution

**Table 3:** Summary of expression status, function, and mechanism of CLKs in different cancer types

Kinase	Cancer type	Expression status	Identified gene function	Phenotype	Mechanism	Ref.
CLK1	Pancreatic ductal adenocarcinoma (PDAC)	Upregulated	Oncogene	Promoted PC cell growth and metastasis in vitro and in vivo Associated with poor prognosis in PDAC	CLK1 enhanced phosphorylation on SRSF5^Ser250^, which inhibited METTL14^exon10^ skipping while promoting Cyclin L2^exon6.3^ skipping to promote cancer cell metastasis and proliferation	^40,44^
	Prostate cancer (PC)	Upregulated	Oncogene	Inhibition of CLK1 decreased cell proliferation and apoptosis in PC3 and DU145 cell lines CLK1 and CLK3 expression was consistently induced in hypoxic conditions in PC3 cells	Alternative splicing in cancer related genes: C *ENPE*, *ESCO2*, *CKAP2*, *MELK*, *ASPH* and *CD164*, which contributed to TG003-inhibited cell growth inhibition by targeting CLK1 CLK1 promoted cancer cell adaption to hypoxia by regulating *CASP9* alternative splicing	^119,120^
	Gastric cancer	Upregulated	Oncogene	CLK1 overexpressed and promoted cell proliferation, migration, and invasion in gastric cancer	CLK1 promoted gastric cancer by modulating its related splicing machinery pathways	^40^
	Ovarian cancer	/	Oncogene	CLK1 phosphorylated SPF45 to elevate SPF45 expression and promote ovarian cancer migration and invasion	CLK1 phosphorylated SPF45 to increase SPF45 expression and promote SPF45-induced exon 6 exclusion of *Fas* mRNA in SKOV3 breast cancer cells	^46^
CLK2	Non-small cell lung cancer (NSCLC)	Upregulated	Oncogene	Promoted NSCLC occurrence and development	CLK2 overexpression promoted NSCLC growth and acted as a biomarker; miR-573 negatively regulated CLK2 expression in NSCLC	^121^
	Breast cancer (BC)	Upregulated	Oncogene	Promoted BC growth and EMT phenotype in luminal breast cancer Pharmacological inhibition of CLK2 decreased cell growth and promoted apoptosis in an allograft model of Myc-driven spontaneous breast cancer	CLK2 overexpression generated high levels of cyclin B1, CDK1, phospho-Rb, and activated hippo signaling pathway CLK2 promoted the EMT variant of ENAH to facilitate breast tumor invasion and metastasis CLK2 inhibition changed the AS of genes involved in cell cycle, DNA repair, RNA splicing, and RNA transport pathways	^42,122^
	Glioblastoma and glioma stem-like cell (GSC)	Upregulated	Oncogene	Depletion of CLK2 expression arrested cell cycle at G1 and S phase	Knockdown of CLK2 downregulated AKT/FOXO3a/p27 signaling to interrupt cell cycle and reduce tumor growth Downregulation of CLK2 decreased the binding affinity of CLK2 with 14–3–3τ, but increased its affinity to phospho-PP2A	^55,123^
CLK3	Cholangiocarcinoma (CCA)	Upregulated	Oncogene	Promoted c-Myc-mediated purine synthesis in CCA	Q607R mutation of CLK3 induced USP13 Y708 phosphorylation, promoted USP13 binding to c-Myc and enhanced c-Myc activity	^124^
	Hepatocellular carcinoma (HCC)	Upregulated	Oncogene	Increased cell proliferation, migration, and invasion in vitro and tumor development in vivo	CLK3 promoted Wnt 3a expression and activated Wnt/β-catenin cascades in HCC	^125^
	Hematopoietic stem cells (HSCs)	/	Oncogene	Reinforced an HSC-specific program	CLK3 effected HMGA2 isoform switching; knock-down of CLK3 decreased HMGA2-S but increased HMGA2-L through SRSF1	^43^
CLK4	MES-TNBC	Upregulated	Oncogene	Promoted MES-TNBC cell invasion, tumor metastasis, and CSC properties	CLK4 promoted MES-TNBC by overexpression or modulation of TGF-β signaling *via* SMAD3	^114^
	ESCC	Downregulated	Suppressor	CLK4 was downregulated in ESCC cells and patient samples. The function of CLK4 in suppressing ESCC development associated with the methylation status of its promoter	CLK4 phosphorylated MITF at Y360 and blocked MITF-enhanced de novo purine synthesis and redox balance	^126^

**Table 4:** Structure and molecular function of CLKs inhibitors

Compound name	Chemical formula	Structure	Targets (IC_50_ or *K*_d_ values)	Administration efficiency		Clinical trial status and efficacy	Ref.
				In vitro	In vivo		
SM08502	C_24_H_25_N_7_O		8 nM on CLK1 2 nM on CLK2 22 nM on CLK3 1 nM on CLK4 2–13 nM on DYRKs 1.1 μM on CDK1	SM08502 inhibited CLKs activity, decreased SRSF phosphorylation, and reduced the generation of splicing variants of Wnt signal pathway genes in gastrointestinal cancer	Oral administration of 6.25 ~ 25 mg/kg SM08502 by QD or QOD inhibited gastrointestinal tumor growth and decreased SRSF phosphorylation in xenograft mouse models	Phase I (NCT03355066) Ongoing Phase I (NCT05084859) Ongoing	^12,127^
TG003	C_13_H_15_NO_2_S		20 nM on mCLK1 200 nM on mCLK2 15 nM on mCLK4 No activity on mCLK3 No activity on SRPK1, SRPK2 and PKC	TG003 reduced cell proliferation, invasion, and migration in gastric and prostate cancer cells TG003 inhibited CLK/STY kinases activities and suppressed phosphorylation of SR proteins in Hela cell line	Intraperitoneal administration of TG003 twice per week decreased prostate cancer CDX growth	NO	^40,47,119,128^
T-025	C_21_H_18_N_8_		0.096 nM on CLK2 4.8 nM on CLK1 6.5 nM on CLK3 0.61 nM on CLK4 0.074 nM on DYRK1 1.5 nM on DYRK1B	T-025 was more effective on cells with high CLK2 expression and Myc-amplification in a dose-dependent manner; facilitating activation of downstream SE events	50 mg/kg T-025 strongly suppressed the growth of breast tumor allograft model	NO	^122^
DB18	C_24_H_18_ClN_7_O_3_		11 nM on CLK1 27 nM on CLK2 1280 nM on CLK3 20 nM on CLK4 120 nM on DYRK1A	DB18 showed high inhibitory effects on MCF-7 and PC3 cell lines; weaker inhibitory effects were observed on fibroblast, HuH7, CaCo-2, MDA-MB-231, HCT116 and NCI-H727 cell lines	NO	NO	^129,130^
CLK1-IN-1	C_24_H_16_FN_5_O		2 nM on CLK1 31 nM on CLK2 8 nM on CLK4 138 nM on DYRK1A	CLK1-IN-1 inhibited CLK1 activation, resulting in the distribution of SR proteins and increasing autophagy and autophagic flux	30 mg/kg CLK1-IN-1 had a hepatoprotective effect by decreasing ALT and AST enzyme levels in an APAP-induced hepatotoxicity mouse model	NO	^104^
CLK-IN-T3	C_28_H_30_N_6_O_2_		0.67 nM on CLK1 15 nM on CLK2 110 nM on CLK3 260 nM on DYRK1A 230 nM on DYRK1B	T3 induced apoptosis and G2/M cell cycle arrest in A2780 and HCT116 cells; synergistically induced apoptosis with Bcl-xL/Bcl-2 inhibitor	NO	NO	^131,132^
KH-CB19	C_15_H_13_Cl_2_N_3_O_2_		19.7 nM on CLK1 530 nM on CLK3 55.2 nM on DYRK1A	KH-CB19 suppressed the phosphorylation of SRp75, SRp55 and SRp20; reduced flTF and asHTF expression; attenuated TNF-α-induced TF mRNA splice variants in HMEC-1 cells	NO	NO	^117^
Cpd-1	C_21_H_20_F_3_N_7_O		16 nM on CLK1 45 nM on CLK2 61 nM on SRPK1 75 nM on SRPK2 10000 nM on SRPK3	Cpd-1, cpd-2, and cpd-3 compounds decreased endogenous phosphorylation of SR proteins and enlarged the nuclear speckles in MDA-MB-468 cells; resulting in splicing alterations of S6K and subsequent S6K protein depletion	NO	NO	^133^
Cpd-2	C_20_H_20_N_6_O		1.1 nM on CLK1 2.4 nM on CLK2 >100 nM on SRPK1/2/3				^133^
Cpd-3	C_21_H_21_N_5_O_2_		1.1 nM on CLK1 2.1 nM on CLK2 >100 nM on SRPK1/2/3				^133^
MU1210	C_22_H_16_N_4_O		8 nM on CLK1 20 nM on CLK2 12 nM on CLK4 >3000 nM on CLK3 29 nM on HIPK2 159 nM on HIPK3 187 nM on HIPK1 213 nM on DYRK1A 956 nM on DYRK1B 1309 nM on DYRK2	MU1210 attenuated MCF-7 cell proliferation and exhibited an IC_50_ of 4.6 μM in cell viability	NO	NO	^134^
Indazole1	C_19_H_24_N_4_O		12 nM on CLK1 10 nM on CLK2 2250 nM on CLK3 12 nM on CLK4 73 nM on DYRK1A	Indazole1 increased the frequency of MNBN in a dose-dependent manner through CLKs inhibition in primary human lymphocytes	NO	NO	^63^
KuWal151	C_16_H_11_ClN_2_O		88 nM on CLK1, 510 nM on CLK2 28 nM on CLK4 Inactive on CLK3, DYRK1A/B and DYRK2 ( > 10 μM)	KuWal151 exhibited less than 0.5 μM potency in more than 50 cancer cell lines, specifically in MDA-MB-435 cell line (GI_50_ = 72.4 nM)	NO	NO	^135^
GPS167	C_17_H_13_N_5_OS		NO	GPS167 impaired cell proliferation and organoids growth of human CRC cells by interrupting the phosphorylation of SRSF10	NO	NO	^45^
Silmitasertib (CX-4945)	C_19_H_12_ClN_3_O_2_		3.8 nM on CLK2 1 nM on CK2α and CK2α‘ 82.3 nM on CLK1 90 nM on CLK3 1.229 μM on SRPK1 1.111 μM on SRPK2	CX-4945 inhibited CLK2 kinase activity and modulated SR protein phosphorylation with an IC_50_ dose between 3 to 90 nM CX-4945 treatment decreased SRSF1, SRSF4, SRSF5, and SRSF6 phosphorylation in 293 T cells	CX-4945 inhibited neoplastic animal models singly or synergistically combined with other inhibitors	Phase I/II (NCT03904862) Recruiting Phase I (NCT03897036) Recruiting Not application (NCT03571438) Recruiting Phase I/II (NCT02128282) Complete	^136^
CC-671	C_28_H_28_N_6_O_4_		6 nM on CLK2 5 nM on TTK 300 nM on CLK1 99 nM on DYRK3 107 nM on DYRK1A 157 nM on DYRK1B 136 nM on PHKG	Luminal breast cancer cells were more sensitive to CC-671 than TNBC cells	NO	NO	^146^
Thiophene 48	C_12_H_8_N_2_S_2_		110 nM on CLK1 100 nM on DYRK1A 70 nM on DYRK1B 40 nM on DYRK2	Thiophene 48 induced cell apoptosis in U2OS osteosarcoma cells at 1 μM by increasing the ratio of caspase 3/7 Thiophene 48 exhibited low toxicity on V79 hamster lung fibroblasts at 5 μM	NO	NO	^147^
Leucettine L41	C_17_H_13_N_3_O_3_		71 nM on CLK1 64 nM on CLK4 60 nM on DYRK1A 44 nM on DYRK1B 73 nM on DYRK2 720 nM on CLK2 >10 μM on CLK3	L41 displayed a neuroprotective role in glutamate induced HT22 cell death L41 treatment significantly elevated the percentage of exon inclusion of the CLK1 minigene by modulating pre-mRNA splicing in Hela cells	NO	NO	^148–150^

*QD* Quaque in Die (every day, once daily), *QOD* Quaque omni die (every other day)

### CLKs in noncancer diseases and therapeutic tactics

#### CLKs in neurodegenerative diseases

Hyperphosphorylation and deposition of tau protein is a hallmark of sporadic Alzheimer’s disease (AD), and the dysregulation of tau exon 10 alternative splicing contributes to neurofibrillary degeneration.^59^ It was also reported that irregular splicing events modulated by CLK1 or CLK2 affected the human adult nervous system contributing to sporadic AD.^4,60,61^ CLK1-mediated phosphorylation of SR proteins at serine residues is a key mechanism that has been implicated in the development of Alzheimer’s disease.^4^ In addition, the processes of tau exon 10 alternative splicing induced by phosphorylating SR proteins, and the pre-mRNA splicing changes caused by mCLK2 tended to be therapeutic concepts of tauopathies, including sporadic AD.^60,62^ Unfortunately, the author of the study did not indicate the exact SRs modulated by mCLK2 in AD. Furthermore, irregular CLK2 expression and activity were observed in AD, which resulting in misregulation alternative splicing of tau exon 10^60^.

Besides, inhibition of CLK2 was proposed to be a potential therapeutic strategy in Phelan-McDermid syndrome (PMDS) by improving autism and neuronal functions.^63^ The loss of ubiquitination modulation of CLK2 expression in *Shank3-deficient* neurons is a genetic hallmark of PMDS. Moreover, TG003 treatment exhibited a similar restorative effect on spine density with CLK2 reduction in *Shank3*- *deficient* neurons.^64^ On the basis that CLK2 is a therapeutic target of PMDS, Indazole 1 and Indazole 2 were shown to increase spine density in mouse brain slices at a relatively low dose (300 nM and 1 µM) compared to TG003 (10 µM).^63^

#### CLKs in Duchenne muscular dystrophy

Duchenne muscular dystrophy (DMD) is a fatal progressive muscle-wasting disease. Based on current research, DMD develops due to open-reading frame disruption or dystrophin mRNA alteration caused by exon deletion or mutations.^65,66^ CLK1-regulated splicing manipulation, especially the exon-skipping function, was reported to contribute to DMD.^67^ A patient harbored c.4303 G > T point mutation in *dystrophin* gene exon 31 changed SRp30c/SRSF9-dependent exon skipping enhancer (ESE) to hnRNP A1-dependent exon skipping silencer (ESS), thus, leading to exon 31 skipping to generate truncated, but functional dystrophin protein. Moreover, TG003, an inhibitor of CLK-1, −2, and −4, promoted *dystrophin* gene exon 31 skipping in patient muscle cells that harbored the c.4303 G > T point mutation (ineffective in exon 31-WT cells) and enabled the generation of partially functional dystrophin protein.^68^ On the other hand, additional SR(s) which could be phosphorylated by CLKs cooperated with hnRNP A1 to recognize exon 31 during splicing after de-phosphorylation by TG003.^68^ TG693, a selective CLK1 inhibitor capable of treating DMD, was discovered to be a more metabolically stable compound relative to TG0003.^67^ Functionally, TG693 mediated SR protein phosphorylation and promoted mutant exon 31 skipping reading on the cells harboring the c.4303 G > T point mutation by targeting CLK1, particularly SRSF4 and SRSF6.^67^ These efforts highlight the potential benefits of using CLKs-targeting compounds for the therapeutic management of genetic diseases characterized by irregular splicing, including DMD.

#### CLKs in inflammatory diseases

Knockdown of CLK2 promoted tenocyte differentiation by inhibiting the expression of tenocyte catabolic enzymes, indicating that targeting CLK2 may play a protective role against tendinopathy.^69^ SM04755 was identified as a potent inhibitor of CLK2 and DYRK1A with an IC_50_ of 5.0 nM and 3.5 nM, respectively. SM04755 potently inhibited CLK2 and DYRK1A to modulate intron retention of Wnt pathway genes, subsequently suppressing Wnt signaling pathway and inhibiting the inflammatory signaling mediators NF-κB and STAT3. This process prevented tendon destruction and promoted tendon regeneration.^69^

Osteoarthritis (OA) is a disease characterized by the formation of osteophytes, cartilage degradation, and synovial inflammation.^70^ CLK2 and DYRK1A participate in OA through the Wnt pathway without modulating β-catenin activity, resulting in inflammatory cytokine production and cartilage catabolic enzyme expression.^71,72^ In addition, CLK2 inhibition suppressed cartilage catabolic enzyme expression and induced early chondrocyte differentiation in human mesenchymal stem cells (hMSCs).^71^ This finding reveals a novel role of CLK2 in promoting OA. Lorecivivint (SM04690) potently inhibited CLK2 (IC_50_ = 7.8 nM) and DYRK1A (IC_50_ = 26.9 nM) to suppress CLK2-mediated phosphorylation of SR proteins and DYRK1A-mediated phosphorylation of SIRT1 and FOXO1. Moreover, Lorecivivint exerted anti-inflammatory effect by significantly decreasing NF-κB and STAT3 activity. Lorecivivint provided both symptom relief and inflammatory modification through dual targeting of CLK2 and DYRK1A.^62^

A Phase I clinical trial (NCT02095548) and phase IIa proof‐of‐concept clinical trial (NCT02536833) among knee OA patients indicated that Lorecivivint appeared to be safe and well-tolerated, emphasizing the therapeutic potential of Lorecivivint in OA.^73,74^ A post hoc analysis from a phase IIb clinical trial (NCT03122860) showed that intra-articular (IA) therapy of 0.07 mg Lorecivivint in participants with knee OA showed long-lasting improvements in function and pain compared to those given placebo.^75,76^ Furthermore, other clinical trials (NCT04385303 (Phase III), NCT03928184 (Phase III), NCT03727022 (Phase II) and) have been carried out and accomplished to reveal the therapeutic potential of Lorecivivint in keen OA.

#### CLKs in viral replication

The HIV-1 virus significantly contributes to the global health burden and the replication of HIV-1 is the key process in the virus life cycle.^77,78^ Substantial efforts have been made to alter RNA splicing of HIV-1, and SR proteins were found to participate in viral replication.^79,80^ Therefore, studies were carried out to investigate the role that CLKs, which are located upstream of SRs, play in HIV-1 replication. CLK1 promoted the expression of HIV-1 Gag, a viral structural protein, while CLK2 significantly decreased Gag expression. However, CLK3 and CLK4 were found to only have modest effects in promoting Gag expression.^81^ Application of chlorhexidine, an inhibitor of CLK2, −3, and −4 (chlorhexidine exhibits minimal activity against CLK2), significantly inhibited HIV-1 Gag synthesis and viral replication. Moreover, chlorhexidine treatment suppressed HIV-1 regulatory protein Rev accumulation to prevent the export of viral RNAs. Moreover, a phase II clinical trial (NCT00006075) was carried out at the year of 2001 to evaluate the best strength of chlorhexidine gluconate in the prevention of mother-to-child transmission (MTCT) of HIV-1 by washing the mother’s vagina and the newborn baby during birth. The trial pointed out that a 1% solution of chlorhexidine exhibited well safety, tolerance, and effectiveness in decreasing the rate of MTCT of HIV-1.^82^ These findings highlight the possibility of targeting host CLKs to block HIV-1 replication.^81^

The life cycle of influenza A also relies on mRNA splicing and splicing-related factors, including CLK1.^83–85^ Knock-down of CLK1 reduced the replication of influenza A/WSN/33 by increasing segment 7 RNA splicing and decreasing viral M1 and M2 proteins, which are essential for influenza virus replication. Furthermore, SRSF3 was identified as a key factor responsible for regulating viral RNA splicing upon CLK1 inhibition. NIH39 and KH-CB19, inhibitors of CLK1, showed antiviral activity with an IC_50_ of 6.6 μM and 13.6 μM, respectively. NIH39 reduced viral mRNA splicing and related protein expression at 12.5 μM. However, KH-CB19 did not show any effect even at relatively high doses of 50 and 100 μM, indicating that NIH39 may be a promising anti-viral therapeutic to combat influenza A.^83^ As CLK1 was found to play a crucial role in influenza replication, screening of CLK1 inhibitors was carried out by a virtual docking program. The virtual docking program indicated that J10688 (clypearin), J12098 (corilagin), and J14848 (pinosylvin) were identified to be the most potential anti-influenza virus candidates as CLK1 inhibitors with EC50 values of 1.2 ± 0.28, 2.0 ± 2.22 and 5.28 ± 2.45 μg/mL, respectively; cytotoxic concentrations (CC50) were >200, 153.54 and 18.26 μg/mL, respectively.^86^ J10688, isolated from Pithecellobium clypearia Benth, showed potent anti-influenza ability through impairing viral proteins NP and M2 synthesis and downregulating the phosphorylation of the splicing factors SF2/ASF and SC35.^87^ These conclusions indicated a novel therapeutic approach that targets host CLKs to suppress viral replication.

Besides HIV-1 and influenza A, CLK1 also plays important roles in the replication of West Nile virus and chikungunya virus, making it an attractive cellular candidate for host-directed antiviral therapy.^88–90^

#### CLKs in autophagy-associated diseases

Autophagy is an important cellular process that protect cells from death and maintain homeostasis through the selective degradation of intracellular hazard.^91^ Alterations in autophagic activity are associated with a wide range of human diseases, including diabetes,^92,93^ cardiovascular diseases,^94,95^ infectious^96,97^ and drug-induced organ injury.^98,99^ CLK1 has been reported to participate in the regulation of the autophagy process. CLK1 inhibition by 10 μM concentration of Leucettine L41 (inhibit both DYRKs and CLKs) treatment was shown to increase LC3 foci formation and induce autophagy in a dose-dependent manner in human osteosarcoma cells U-2 OS.^100^ Meanwhile, CLK1 RNA interference in U-2 OS cells resulted in elevated cell autophagy. However, Leucettine L33 (inactive on both CLK1 and DYRKs) and L38 (more potent to DYRKs) were unable to trigger autophagy in U-2 OS cells.^100^ The findings highlight that CLKs inhibition contribute more to autophagy than DYRKs. Mechanistically, L41 treatment significantly elevated exon 4-containing CLK1 mRNA transcripts resulting in an increase of the full-length kinase isoform by modulating its own splicing.^100,101^ Additionally, the targeting of CLK1 by inhibitors or siRNA reagents was speculated to trigger autophagy through modulating the alternative splicing of autophagy regulator pre-mRNAs, including mTOR and X-box–binding protein 1 (XBP1).^100,102,103^ These findings further confirm that CLK1 inhibitors are potent inducers of autophagy. Moreover, compound 9e, a drug derived from 3,6-disubstutited-imidazo [1,2-α] pyridine, potently inhibited CLK1 with an IC_50_ value of 4 nM compared with CLK2 (IC_50_ of 50 nM) and CLK4 (IC_50_ of 17 nM). Treatment with compound 9e induced autophagy, significantly increased autophagic flux, and affected the subcellular redistribution and phosphorylation levels of the downstream SR proteins of CLK1 in SKOV-3 cells.^91^ In addition, CLK1-IN-1 was shown to induce autophagy by targeting CLK1 in the treatment of autophagy-associated diseases. The downstream SR proteins of CLK1 were redistributed from the nucleoplasm to nuclear speckles after CLK1-IN-1 treatment. Furthermore, CLK1-IN-1 induced autophagy in BNLCL.2 and SKOV-3 cells by elevating LC3II protein expression and increasing the ratio of LC3II to LC3I in a dose- and time-dependent manner by targeting CLK1.^104^ Collectively, inhibition of CLK1 increased cellular autophagy, indicating its potential as a treatment strategy for autophagy-associated diseases.

#### CLKs in other diseases

Pathological cardiac hypertrophy is characterized by hypertrophic growth and cardiomyocyte size increase in response to pathological stimuli.^105,106^ CLK4 was found to play a pivotal role in maintaining cardiac function; the deletion of CLK4 contributed to pathological myocardial hypertrophy and heart failure.^56^ Phosphorylation of NEXN at Ser437 by CLK4 was found to rescue pathological myocardial hypertrophy, which highlights the splicing-independent involvement of CLK4 in heart disease. Moreover, the finding indicated that restoration of CLK4 may act as a novel therapeutic target for treating pathological cardiac hypertrophy.^56^

Hepatic gluconeogenesis is critical for hyperglycemia in diabetes.^107,108^ CLK2 was identified as a suppressor of hepatic gluconeogenesis, and its activity was regulated by insulin/AKT.^50^ Insulin/AKT stabilized CLK2 and increased its expression and activity during the feeding/fasting nutrient cycle. Mechanistically, Insulin treatment led to CLK2 phosphorylation at Ser342/Thr343 in H2.35 hepatocytes. Moreover, AKT specifically interacted with CLK2 at its kinase domain and phosphorylated CLK2 at Thr343 in response to insulin. Activated CLK2 phosphorylated PGC-1α at its SR domain to repress PGC-1α transcriptional activity on gluconeogenic genes, resulting in hepatic gluconeogenesis suppression.^50^

Furthermore, CLKs contributed to the sensing of temperature differentials through the phosphorylation of SR proteins.^109^ CLK1/4 became completely inactivated at 38 °C and was re-activated at 35 °C, indicating the potential role of CLK1/4 as molecular thermometers in response to circadian body-temperature oscillations.^109,110^ TG003 application dramatically abolished the re-phosphorylation of SR proteins, such as SRSF5 and SRSF6, during the temperature shift from 42 °C to 35 °C.^109^ Meanwhile, temperature-dependent AS events, including exon inclusion and intron retention, were modulated after TG003 treatment, illustrating the pivotal role of CLK1/4 in connecting body temperature with AS.^109^

To summarize, irregular splicing or expression of CLKs involved in various human diseases, for instance, Alzheimer’s disease, osteoarthritis, Duchenne muscular dystrophy, pathological cardiac hypertrophy, and gluconeogenesis. Moreover, inhibitors have been reported to have anti-inflammatory, anti-viral, and anti-neurodegenerative functions as well as other functions. Nevertheless, the clinical application of these inhibitors is in great demand which may provide direct evidence for the therapy of different human diseases.

### CLKs in cancer and treatment strategies

Expression or activities change of CLKs are associated with cancer development and progression.^111–115^ Depletion or chemical inhibition of CLKs changed alternative splicing events leading to decreased cell proliferation.^67,116–118^ Given the expression status and clinical significance of CLKs in The Cancer Genome Atlas Program (TCGA) database (https://portal.gdc.cancer.gov/), we systematically discuss the roles of CLKs in cancer and therapeutic effects of their related inhibitors (Table 3 and Figs. 5, 6).

#### CLK1 in cancer

The expression of CLK1 was dramatically increased in pancreatic ductal adenocarcinoma (PDAC) tissues at both the mRNA and protein levels, promoting cell growth and metastasis in vitro and in vivo.^44^ High expression of CLK1 was associated with poor prognosis in PDAC. Mechanistically, CLK1 was found to directly phosphorylate SRSF5 at Ser250. Consequently, this inhibited METTL14^exon10^ skipping (generated METTL14-L isoform) to enhance N6-methyladenosine (m6A) methylation and cancer metastasis. Additionally, Cyclin L2^exon6.3^ skipping (generated CCNL2-S isoform) was found to promote PDAC cells proliferation in response to SRSF5 phosphorylation at Ser250.^44^ These results revealed a therapeutic option and potential prognostic value of CLK1/SRSF5 pathway in PDAC patients.

Inhibition of CLK1 decreased cell proliferation and induced apoptosis in PC3 and DU145 prostate cancer cells; the responsive AS events were validated by transcriptomic analysis.^119^ The sequencing revealed the dramatic change of alternative splicing events in the following cancer related genes: centromere protein E (*CENPE*, inclusion at exon 38), establishment of sister chromatid cohesion N-acetyltransferase 2 (*ESCO2*, skipping at exon 9), cytoskeleton associated protein 2 (*CKAP2*, skipping at exon 3), maternal embryonic leucine zipper kinase (*MELK*, skipping at exon 13), aspartate β-hydroxylase (*ASPH*, skipping at exon 6 and 8) and *CD164* (skipping at exon 5).^119^ In hypoxic conditions, CLK1 and CLK3 expression were consistently induced in PC3 cells exposed to 1%, 0.2%, and 0% hypoxic environments; changes in *CASP9* splicing were also observed.^120^

CLK1 was expressed at levels 10 to 30% higher in gastric tumor samples compared to normal gastric tissues.^40^ In a phosphor-proteomic analysis study, CLK1 and its related splicing machinery pathway were shown to be the most important regulators in gastric cancer.^40^ CLK1 was overexpressed in gastric cancer tissues and the inhibition of CLK1 resulted in the suppression of cell proliferation, migration, and invasion.^40^ These findings indicate that CLK1 plays an important role in gastric cancer progression.

SPF45 was identified to be a cellular target of CLK1 in ovarian cancer.^46,115^ (LC)-electrospray ionization-tandem mass spectrometry (MS/MS) experiments showed that CLK1 is able to phosphorylate SPF45 at eight serine residues (Ser 48, 62, 202, 204, 222, 266, 288, and 291).^46^ Furthermore, CLK1 overexpression increased SPF45 protein levels and promoted SPF45-induced exon 6 exclusion of *Fas* mRNA in SKOV3 breast cancer cells. Additionally, inhibition of CLK1 decreased the half-life of SPF45 via a proteasome-dependent manner in both SKOV-3 and HeLa cells. Interestingly, kinase dead Ser 48/222/266 mutants significantly increased exon 6 exclusion while Ser 202/204 A significantly decreased exon 6 exclusion compared with wild-type Myc-SPF45 in COS-1 cells. The finding further illustrates that CLK1 differentially regulates SPF45 splicing activity depending upon phosphorylation at different serine sites. Moreover, SPF45 enhanced fibronectin expression to promote ovarian cancer migration and invasion in a CLK1 phosphorylation-dependent way.^46^ This finding highlights the potential use of CLK1 inhibitors to dephosphorylate SPF45 in ovarian cancer treatment.

Based upon data provided by TCGA, CLK1 is significantly overexpressed in many cancer types, including cholangiocarcinoma (CHOL), colon adenocarcinoma (COAD), head and neck squamous cell carcinoma (HNSC), kidney renal clear cell carcinoma (KIRC), kidney renal papillary cell carcinoma (KIRP), liver hepatocellular carcinoma (LIHC), prostate adenocarcinoma (PRAD), rectum adenocarcinoma (READ) and stomach adenocarcinoma (STAD) (Fig. 5a). In addition, CLK1 was considered to participate in the onset, progression, and evolution of cancers through different mechanisms, however, the function probably was cancer specific.

#### CLK2 in cancer

Increased expression of CLK2 was demonstrated to be correlated with poor prognosis in non-small cell lung cancer (NSCLC).^121^ In addition, CLK2 expression was elevated in patients with late-stage (III–IV) cancer and metastasis, indicating that CLK2 could be a potential biomarker in NSCLC. Moreover, miR-573 suppressed CLK2 expression and interrupted the occurrence and development of lung cancer caused by CLK2 overexpression.^121^

In luminal breast cancer, overexpression and amplification of CLK2 were demonstrated to promote cell proliferation, migration, invasion, and xenograft growth.^42^ Breast cancers which harbored high CLK2 expression levels showed a more proliferative phenotype based on increased levels of cyclin B1, CDK1, phospho-Rb, and the activation status of hippo signaling pathway.^42^ Moreover, CLK2 was found to promote the EMT variant of ENAH to facilitate breast tumor invasion and metastasis.^42^ Pharmacological inhibition of CLK2 resulted in significant growth inhibition, apoptosis, and exon skip in the allograft model of Myc-driven spontaneous breast cancer.^122^ RNA-seq analysis also revealed that genes involved in cell cycle, DNA repair, RNA splicing, and RNA transport pathways were modulated in alternative splicing, further indicating the potential role of AS by CLK2 in breast cancer.^122^

The cell cycle of glioblastoma cell lines with elevated CLK2 levels was arrested at G1 and S phases as a consequence of CLK2 depletion.^123^ Mechanistically, knockdown of CLK2 was shown to interrupt the cell cycle in vitro through the downregulation of AKT/FOXO3a/p27 signaling, resulting in reduced glioblastoma tumor growth and prolonged survival in vivo.^123^ Moreover, CLK2 expression in glioblastoma patient specimens was inversely correlated with patient survival time.^123^ In addition, 14–3–3τ directly bond with CLK2 to increase CLK2 stability through modulating proteasomal degradation in glioma stem-like cells (GSCs).^55^ Meanwhile, CLK2 negatively regulated PP2A activity in the GSC272 brain tumor cancer cell line. Downregulation of CLK2 led to decreased binding affinity with 14–3–3τ and increased binding affinity with phosphor-PP2A, resulting in the activation of PI3K signaling pathway. Thus, the combination of CLK2 depletion with the PI3K/mTOR inhibitor GSK2126458 significantly reduced tumor growth in GSC272-implanted mice. Furthermore, the induction of apoptosis was also observed when FGFR inhibitor (LY2874455) was administered to a CLK2 knockdown GSC mouse model.^55^

RNA-Seq data provided by the TCGA has shown that CLK2 is overexpressed in many cancer types (Fig. 5a). Increased expression of CLK2 was correlated with poor clinical outcomes in cervical squamous cell carcinoma and endocervical adenocarcinoma (CESC), COAD and KIRC (Fig. 5b). In conclusion, CLK2 might be an ideal therapeutic target and prognostic marker for cancer treatment.

#### CLK3 in cancer

CLK3 was found to be upregulated in cholangiocarcinoma (CCA) patients and plays a role in nucleotide metabolism.^124^ Meanwhile, a gain of function somatic mutation Q607R was identified in CLK3 kinase domain, which induced USP13 Y708 phosphorylation and promoted USP13 binding to c-Myc. The binding was found to prevent c-Myc ubiquitination and enhance c-Myc activity, thereby, increasing c-Myc-mediated purine synthesis in CCA.^124^ In turn, a CLK3-USP13-c-Myc feedback loop was identified whereby activated c-Myc increased CLK3 transcription by enhancing CLK3 promoter activity. However, the other CLKs family members were not affected by c-Myc.^124^

CLK3 was also markedly upregulated and closely associated with hepatocellular carcinoma (HCC) TNM stages and patient prognosis.^125^ Functional analysis revealed that CLK3 promoted Wnt 3a transcription and activated Wnt/β-catenin cascades, resulting in increased HCC cell proliferation, migration, and invasion in vitro*;* additionally, animal experiments showed that CLK3 also increased tumor development in vivo. Moreover, the miR-144/CLK3 axis was found to further attenuate Wnt/β-catenin signaling, resulting in suppression of HCC development and metastasis.^125^

CLK3 also affected HSCs by modulating HMGA2 alternative splicing.^43^ Mechanistically, CLK3 strongly affected HMGA2 isoform switching; knock-down of CLK3 decreased HMGA2-S transcription but increased HMGA2-L transcription through SRSF1. The results indicated that CLK3 was involved in regulating an SRSF1-dependent splicing pattern that enhanced the development of human HSC.^43^

Summarization from the TCGA database demonstrated that elevated expression of CLK3 was observed in CHOL, HNSC, Kidney chromophobe (KICH), KIRC, KIRP, LIHC, PRAD, and STAD. Furthermore, increased expression of CLK3 was correlated with poor overall survival (OS) outcome in COAD, indicating the clinical significance of CLK3 (Fig. 5). Thus, targeting CLK3 by siRNA or antagonists could be taken into consideration for further investigation.

#### CLK4 in cancer

CLK4 was overexpressed in mesenchymal-like TNBC (MES-TNBC) cells and correlated with poor patient survival.^114^ Silencing of CLK4 in a xenograft mouse model was shown to decrease the expression of multiple epithelial-mesenchymal transition (EMT) genes which participate in metastasis and repress tumor cell migration in TNBC cells.^114^ Notably, depletion of CLK4 impaired the expression of SMAD3, a mediator of TGF-β signal transduction, suggesting that overexpression of CLK4 can promote metastatic and aggressive phenotypes in MES-TNBC cells. Furthermore, the pharmacological inhibition of CLK4 suppressed the growth and invasiveness of MES-TNBC cells, highlighting the potential utilities of CLK4 in the clinic.^114^

CLK4 was also found to be extensively downregulated in esophageal squamous cell carcinoma (ESCC) cells and patient samples due to the methylation of its promoter.^126^ Mechanistically, CLK4 phosphorylated microphthalmia-associated transcription factor (MITF) at Tyr360 to promote the autophagy degradation of MITF. As a feedback axis, MITF bond to the E-boxes in CLK4 promoter transcriptionally downregulating the expression of CLK4 in ESCC. Moreover, CLK4 was proved to be a redox-sensitive kinase. Interestingly, impairment of CLK4 kinase activity upon oxidation of Met307 was found to enhance ESCC carcinogenesis.^126^ This finding revealed a novel function of CLK4 in modulating purine synthesis and redox status in conjunction with its role in alternative splicing.

CLK4 expression varied in different cancer datasets, among which the expression was significantly elevated in CHOL, glioblastoma multiforme (GBM), HNSC, KIRC, KIRP, LIHC, PRAD, and STAD; Conversely, CLK4 transcription was reduced in BLCA, breast invasive carcinoma (BRCA), lung squamous cell carcinoma (LUSC), pheochromocytoma and paraganglioma (PCGC), thyroid carcinoma (THCA) and uterine corpus endometrial carcinoma (UCEC) (Fig. 5).

The frequency of elevated CLKs expression in gastric, prostate, lung, and cholangiocarcinoma patient tumor tissues^40,42,119–121,124^ suggests that CLKs may directly or indirectly contribute to tumor development, progression, or metastasis. Besides, mutation (CLK3)^124^ or post-translational modification (CLK4) of CLK,^126^ alteration of CLKs expression or function influenced tumor phenotypes. Therefore, high throughput sequencing and chemical development based on mutation sites might be an effective method to highlight clinically relevant molecular features of CLKs.

#### Targeting CLKs for cancer therapeutic strategies

The large body of evidence provided in this review has illustrated the major oncogenic function of CLKs in cancer. Thus, targeting CLKs has garnered increased attention from clinical researchers as a potential method of treating several cancer types. A number of inhibitors that generally bind in the CLKs ATP pocket have been extensively studied and reported (Table 4, Figs. 1b and 6).

SM08502 (Cirtuvivint) is the first small molecular inhibitor of CLKs which underwent clinical trials. SM08502 is a potent pan-CLKs inhibitor and showed strong affinity for CLKs with IC_50_ values of 8 nM to CLK1, 2 nM to CLK2, 22 nM to CLK3, and 1 nM to CLK4.^127^ Although SM08502 showed high affinity to CLKs, it inhibited the activity of other structurally similar kinases as well, indicating off-target effects and the potential for cytotoxicity.^12,127^ SM08502 was shown to significantly inhibit the kinase activity of CLKs and decrease SRSF phosphorylation in gastrointestinal cancers. Furthermore, SM08502 disrupted spliceosome activity, thus, reducing the generation of splicing variants of Wnt signal pathway genes.^127^ Oral administration of SM08502 significantly inhibited the growth of gastrointestinal tumors. SM08502 also decreased SRSF phosphorylation and Wnt pathway gene expression in xenograft mouse models, suggesting that SM08502 is a potent therapeutic drug in cancer treatment.^127^ A Phase I clinical trial of SM08502 (NCT03355066) assessing its efficacy in treating advanced solid tumors for whom no standard therapy is currently underway. This trial aimed to evaluate the safety, tolerability, PK, PD, and preliminary anti-tumor efficacy of SM08502 by oral administration. Additional clinical trials (NCT05084859) for castration-resistant prostate cancer, non-small cell lung cancer, and colorectal cancer were posted in 2021 to evaluate the safety, tolerability, PK, and preliminary anti-tumor efficacy of SM08502.

TG003, a benzothiazole compound, was found to have a potent inhibitory effect on CLK1 and CLK4 *via* in vitro phosphorylation assay.^128^ The activities of TG003 on different CLKs and other kinases, including SRPKs and PKC, were evaluated. Results showed strong inhibitory activity of TG003 on murine CLKs (mCLKs) with IC_50_ values of 20 nM on mCLK1, 200 nM on mCLK2, and 15 nM on mCLK4; however, TG003 showed less inhibitory activity against mCLK3, SRPK1, SRPK2, and PKC kinases.^128^ Moreover, TG003 acted on CLK1/Sty kinase competitively with ATP (K_m_ 3.35 μM) with a Ki value of 10 nM.^128^ In addition, 10 μM TG003 inhibited Clk/Sty kinase activity and SR protein phosphorylation in Hela cell.^128^ TG003 was also found to inhibit CLK-dependent alternative splicing and serine/arginine-rich protein phosphorylation.^128^ The application of TG003 significantly decreased gastric cancer cell viability, invasion, and migration. Similar inhibition effects were found in gastric cancer after depletion of CLK1 by small interfering RNA (siRNA).^40^ Mechanistically, TG003 treatment led to the decrease of splicing protein pSRPK2, SRSF2, CLK1, and p-AKT.^40^ In prostate cancer (PC), TG003 reduced cell proliferation, induced apoptosis, and reversed EMT markers in vitro*;* in vivo CDX tumor growth was also decreased upon treatment with TG003.^119^ In addition, TG003 was found to regulate the alternative splicing of CLK1 mRNA by reducing intron 4 retention and exon 4 skipping, resulting in altered production of full-length catalytically active (CLK1^T1^) and truncated catalytically inactive (CLK1^T2^) isoforms.^47^

T‐025, a chemical modified from the 7*H*‐pyrrolo [2,3‐*d*] pyrimidine structure, was developed as a potent inhibitor of CLK2 able to bind within the ATP-binding pocket and interact with the Glu244 and Leu246 amino acid residuess.^122^ The *K*_d_ values of T-025 to CLK1, -2, -3, -4 were 4.8, 0.096, 6.5, and 0.61 nM, respectively; low *K*_d_ values to DYRKS family proteins were also observed.^122^ As a highly potent inhibitor to CLK/DYRK1, T-025 exhibited more than 300-fold enhanced selectivity compared with other kinases during a KINOME*Scan*-based kinase profiler assay.^122^ T-025 was more sensitive to the cells with elevated CLK2 expression and Myc-amplification in a dose-dependent manner.^122^ Additionally, 50 mg/kg T-025 strongly suppressed the growth of a breast tumor allograft model suggesting that T-025 exerts anticancer effects against Myc-driven breast cancers.^122^

DB18 is a potent inhibitor of CLK1, -2, and -4 kinases belonging to the nilino-2-quinazoline derivatives.^129^ DB18 attenuated CLKs kinase activities with IC_50_ values of 11 nM on CLK1, 27 nM on CLK2, 1280 nM on CLK3, and 20 nM on CLK4 based on radiometric γ^33^P-ATP assay.^129^ When screening at 10 μM concentration with 10 μM ATP, the compound also showed slight affinity to DYRK1A at 120 nM.^129,130^ Surprisingly, high DB18 concentrations (100 μM) showed no toxicity on the activity of both human and rat DYRK1A, human DYRK1B, and DYRK2. Therefore, DB18 may be considered a novel and promising CLKs selective inhibitor.^129^ A cytotoxicity screening assay of DB18 was carried out in different cancer cell lines and normal human fibroblast.^129^ The data demonstrated that DB18 exhibited potent cytotoxicity on MCF-7 and PC3 cell lines with IC_50_ values of 4 and 7 μM. Moreover, DB18 showed moderate cytotoxicity on fibroblast and HuH7 at 21 and 25 μM, and weak activity on CaCo-2, MDA-MB-231, HCT116, and NCI-H727 with IC_50_ higher than 25 μM.^129^ The variability of DB18 efficacy across cell lines is likely dependent on the CLKs expression levels and kinase activities.

CLK1-IN-1 was designed as a potent and selective inhibitor of CLK1 with an IC_50_ of 2 nM. The activity of CLK1-IN-1 against its targets is highly dependent on the residues comprising the kinase domains.^104^ CLK1-IN-1 strongly suppressed CLK1 kinase activity with 69-fold higher in activity compared to the inhibitory effect of CLK1-IN-1 against DYRK1A (IC_50_ = 138 nM).^104^ CLK1-IN-1 was also shown to inhibit other CLKs activities with IC_50_ values of 31 nM to CLK2 and 8 nM to CLK4.^104^ Treatment of BNL CL.2 (mouse embryonic liver cell) with 10 μM CLK1-IN-1 significantly inhibited CLK1 activation, resulting in the redistribution of SR proteins from the nucleoplasm to nuclear speckles, and an increase of autophagy and autophagic flux in vitro in a dose-dependent manner.^104^ Meanwhile, CLK1-IN-1 elevated LC3II expression and induced autophagy and autophagic flux in a dose- and time-dependent manner in the SKOV-3 human ovarian cancer cell line, indicating its therapeutic potential for treating certain cancers.^104^ Furthermore, CLK1-IN-1 (30 mg/kg) was reported to have a hepatoprotective effect by decreasing alanine aminotransferase (ALT) and aspartate aminotransferase (AST) expression levels in an Acetaminophen (APAP)-induced hepatotoxicity mouse model.^104^

CLK-IN-T3 exhibited dramatic inhibitory activity against CLKs with IC_50_ values of 0.67 nM for CLK1, 15 nM for CLK2, and 110 nM for CLK3, respectively. However, the change in CLK4 activity post-T3 treatment was not directly measured in that work.^131^ DYRK1A and DYRK1B, dual specificity kinases of the CMGC sub-family, were inhibited 200–300 times less efficiently than CLKs based on kinase enzymatic assays.^131^ The results of RNA-Seq analysis indicated that distinct RNA-binding motifs in skipped exons were associated with T3 treatment.^131^ Meanwhile, T3 application decreased the phosphorylation of the SR proteins, which are located downstream of CLKs. T3 induced apoptosis and G2/M cell cycle arrest in human A2780 and HCT116 cells by targeting CLK.^131^ Mechanically, T3 application modified AS events in cancer by decreasing the expression of the anti-apoptotic forms of cIAP1, cIAP2, XIAP, cFLIP, and Mcl-1.^132^ Meanwhile, T3 synergistically induced apoptosis together with Bcl-xL/Bcl-2 inhibitor in human HCT116 and A2780 cancer cells.^132^

KH-CB19 is a potent and highly selective CLKs inhibitor that was demonstrated to strongly bind with CLK1 and CLK4 in temperature shift assays.^117^ Further enzymatic assay revealed a relatively lower IC_50_ on CLK1 compared with IC_50_ values of 530 nM on CLK3 and 55.2 nM on DYRK1A.^117^ KH-CB19 was revealed to bind with the ATP-binding sites of CLK1 and CLK3 based on co-crystal structures. Further experiments demonstrated that 10 μM KH-CB19 suppressed the phosphorylation of SRp75, SRp55, and SRp20 compared to TG003, which only inhibited SRp20 phosphorylation. Additionally, 10 μM KH-CB19 was able to inhibit full-length tissue factor (flTF) and alternatively spliced human tissue factor (asHTF) expression in HMEC-1 cells.^117^ KH-CB20, an *E/Z*-mixture compound, shared the same structure and similar kinase binding affinity with *E*-isomer KH-CB19 with IC_50_ values of 16.5 nM on CLK1, 488 nM on CLK3 and 57.8 nM on DYRK1A.^117^ These results illustrated the potential of KH-CB19 and KH-CB20 as lead compounds for further drug development.

Cpd-2 and cpd-3 possessed high affinity to CLK1/2 compared to SRPK1/2/3 with IC_50_ values of 1.1 nM (both cpd-2 and cpd-3), 2.4 nM (cpd-2) or 2.1 nM (cpd-3) on CLK1/2, while showed more than 100 nM activity dose (both cpd-2 and cpd-3) on SRPK1/2/3, separately.^133^ In comparison, the IC_50_ values of cpd-1 are 16 and 45 nM on CLK1 and CLK2; the IC_50_ values of SRPK1, -2, and -3 were calculated as 61, 75, and 10000 nM. Cpd-1, cpd-2, and cpd-3 significantly decreased endogenous phosphorylation of SR proteins and enlarged the nuclear speckles in MDA-MB-468 cells. Meanwhile, the inhibitors resulted in splicing alterations of *RPS6KB1*(S6K) and subsequently caused S6K protein depletion.^133^ Moreover, cpd-2 and cpd-3 showed considerable growth inhibition (GI_50_) values in different cancer cell lines: for GI_50_ of cpd-2, 3.0 μM on breast cancer (MDA-MB-468), 1.9 and 1.4 μM on NSCLC (A549 and NCI-H23) and 1.7, 2.2, 2.0 and 0.6 μM on colorectal cancer (COLO205, HCT116, SW620, and COLO320DM); for GI_50_ of cpd-3, 3.4 μM on breast cancer (MDA-MB-468), 2.6 and 2.2 μM on NSCLC (A549 and NCI-H23) and 2.1, 2.5, 2.9 and 1.5 μM on colorectal cancer (COLO205, HCT116, SW620, and COLO320DM). The data suggested that cpd compounds functioned as CLK inhibitors that exerted tumor growth inhibitory effects through splicing alterations.^133^

MU1210 is a potent inhibitor of CLKs with IC_50_ values of 8 nM for CLK1, 20 nM for CLK2, 12 nM for CLK4, and more than 3000 nM for CLK3; notably, off target against HIPKs and DYRKs were observed at relatively high IC_50_ concentration.^134^ MU1210 attenuated MCF-7 cell proliferation and showed IC_50_ of 4.6 μM in cell viability.^134^ However, the detailed biological functions and potential of MU1210 in cancer treatment are largely uncharacterized.

Indazole1 is a novel potent in-house inhibitor of CLK2 with an IC_50_ of 10 nM that was identified by in silico screening.^63^ Indazole1 also potently inhibited CLK1, CLK3, CLK4, and DYRK1A in vitro with the IC_50_ values of 12 nM, 2250 nM, 12 nM, and 73 nM, respectively. Of note, the data demonstrated that Indazole1 tended to be a more potent CLKs inhibitor than TG003 by increasing the frequencies of micro-nucleated binucleates (MNBN) in a dose-dependent manner in primary human lymphocytes.^63^

KuWal151, a member of 3-Aryl-substituted 6,7-dihydropyrrolo[3,4-g]indol-8(1H)-ones class, was identified as an inhibitor of CLK1, -2 and -4 with the IC_50_ values for CLK1/2/4 of 88 nM, 510 nM and 28 nM, respectively.^135^ Interestingly, this compound is inactive against CLK3, DYRK1A/B, and DYRK2.^135^ KuWal151 showed less than 500 nM potency in more than 50 cancer cell lines, especially in the MDA-MB-435 cell line (GI_50_ = 72.4 nM) which exhibited heightened expression of CLK1.^135^ UACC-257 cells showed the lowest CLK1 expression levels and were least sensitive to KuWal151 application with a GI value over 50 μM.^135^ The sensitivities of different cancer cell lines with distinct expression levels of SPF45, an essential substrate of CLK1, to KuWal151 were determined. The results indicated that cells (HCT-116, HCT-15, HT-29, KM12, MCF-7) with increased SPF45 levels were dramatically suppressed by KuWal151. In contrast, OVCAR-8, HOP-92, and MDA-MB-431 were less sensitive to the compound under similar conditions, further proving the correlation between KuWal151 and CLK1.^135^ Thus, KuWal151 might be a potential compound or suitable lead compound for the synthesis of anti-cancer agents targeting CLKs.

GPS167 is a novel CLKs inhibitor that originated from a class of compounds to interrupt HIV replication. GPS167 was subsequently identified to exhibit approximately 50% inhibition on splicing response at 2 μM.^45^ A ^32^P-kinase assay revealed that GPS167 decreased the CLKs-mediated phosphorylation of SRSF10, but not by DRPK1 and SRPK, in a dose-dependent manner. The Colo205, SW620, and HCT116 cancer cell lines were most sensitive to GPS167 as evidenced by the results of CellTox-Green assays. However, the compound showed less cytotoxicity on CRL-1831, CRL-1790, and Caco-2 normal colonocyte cell lines.^45^ Intriguingly, the inhibition of GPS167 on cell growth and viability was discovered to be p53-dependent.^45^ Collectively, GPS167 is a novel inhibitor of CLKs which impairs cell proliferation and organoids growth of human CRC cells *via* interrupting SRSF10 phosphorylation.^45^

Silmitasertib (CX-4945) is a dual inhibitor of CLK2 and CK2. CX-4945 strongly inhibited CK2 with an IC_50_ value of 1 nM against CK2α and CK2α‘; however, it also had a high affinity for CLKs and DYRK1A.^136^ The reported IC_50_ value of CX-4945 on CLK1 is 82.3 nM, on CLK2 is 3.8 nM, on CLK3 is 90 nM, and more than 1000 nM on SRPK1 and SRPK2.^136^ CX-4945 was shown to be a CLK2 ATP-competitive inhibitor capable of modulating SR protein phosphorylation with an IC_50_ concentration ranging between 3 to 90 nM.^136^ The phosphorylation status of SRSF4, SRSF6, SRSF5, and SRSF1 were profoundly decreased in 293-T cells following CX-4945 treatment, indicating that CLKs are targets of the inhibitor.^136^ Surprisingly, CX-4945 showed a greater inhibitory effect than TG003, with a comparable effect on SR protein phosphorylation observed at 1 µM CX-4945 and 10 µM TG-003, suggesting the potential for treating diseases characterized by splicing dysregulation.^136^ The efficacy of CX-4945 has been demonstrated in a broad range of human malignancies. CX-4945 inhibited cell proliferation and induced caspase-3 independent non-autophagic cell death in CCA Cells^137^; CX-4945 suppressed TGF-β1-induced migration and invasion in human A549 cancer cells,^138^ and arrested cell cycle in vitro Moreover, CX-4945 inhibited BT-474 and BxPC-3 xenograft tumor growth in vivo in a dose-dependent way.^139^ Moreover, a synergistic effect was observed when CX-4945 was combined with bortezomib to treat acute lymphoblastic leukemia^140^ and multiple myeloma and mantle cell lymphoma cell lines.^141^ Increased therapeutic effects were observed when CX-4945 cooperated with dasatinib in ovarian cancer;^142^ cooperated with cisplatin and gemcitabine in holangiocarcinoma^143,144^ and with temozolomide in GBM.^144,145^

A phase I/II clinical trial (NCT03904862) of CX-4945 that aims to test the safety and tolerability in individuals with recurrent medulloblastoma is currently recruiting patients. Another phase I clinical trial (NCT03897036) to evaluate the treatment duration and pharmacodynamics of CX-4945 in basal cell carcinoma (BCC) is also currently recruiting volunteers. Additionally, a combination study of CX-4945 with ATM inhibitors (Sunitinib, Pazopanib, and Temsirolimus) in kidney cancer (NCT03571438) which aims to evaluate the therapeutic potential of the compounds in clinical treatment is currently ongoing. A recently completed phase I/II clinical trial (NCT02128282) estimated the safety and tolerability of increasing doses of CX-4945 in combination with gemcitabine plus cisplatin to determine the maximum tolerated dose (MTD) and the recommended Phase II dose (RP2D) in the frontline treatment of patients with cholangiocarcinoma. 200 mg CX-4945 combined with 25 mg/m.sq. cisplatin or 1,000 mg/m.sq. gemcitabine was adopted in MTD and RP2D estimation. Unfortunately, the results are presently unavailable to the public. Additional experiments and clinical trials are currently scheduled to assess the therapeutic potential of CX-4945 in cancer treatment.

CC-671 is a dual inhibitor of CLK2 and Monopolar spindle 1 (Mps1, also named TTK).^146^ Seven kinases showed over 80% inhibition by CC-671 across a 255 kinases panel. TTK and CLK2 activities were potently suppressed of the seven identified kinases with IC_50_ values of 5 nM and 6 nM, respectively.^146^ Other kinases, such as CLK1 or DYRKs, were also inhibited by CC-671 with different IC_50_ values.^146^ Interestingly, results from a series of ActivX KiNative™ profiling assays indicated that CLK2 was inhibited in vitro by CC-671 with an IC_50_ of 15 nM; however, TTK was not inhibited.^146^ Furthermore, treatment of different breast cancer cell lines with CC-671 showed that luminal BC cells were more sensitive to CC-671 treatment versus TNBC cells.^146^

Thiophene 48 is a dual DYRK/CLK1 inhibitor that was synthesized based on the natural molecule harmine.^147^ Thiophene 48 showed potent activity with IC_50_ values of 110 nM, 100 nM, 70 nM, and 40 nM on CLK1, DYRK1A, DYRK1B, and DYRK2, respectively. Moreover, the compound was found to have a 75-fold, 3-fold, and 2-fold better inhibitory activity against DYRK2, DYRK1B, and CLK1 compared to the reference compound harmine.^147^ 1 μM Thiophene 48 significantly induced cell apoptosis in U2OS osteosarcoma cells by increasing the ratio of caspase 3/7. However, Thiophene 48 showed no cytotoxicity at 5 μM on V79 hamster lung fibroblasts while the same concentration of Harmine significantly inhibited cell growth.^147^

Leucettine L41 is a dual inhibitor of CLK1/4 and DYRKs. Leucettine L41 was modified by using Leucettamine B as an inhibitory scaffold, and was shown to co-crystallized with DYRK1A, DYRK2, CLK3, PIM1, and GSK-3β.^148,149^ Leucettine L41 exhibited potent inhibition on DYRK1A, DYRK1B, and DYRK2 with IC_50_ values of 60, 44, and 73 nM, respectively. However, the IC_50_ values with respect to CLKs are much higher.^148^ Compared to the effect of leucettamine B, the inhibitory activities of L41 were significantly increased against DYRKs, CLK1, and CLK4; however, the IC_50_ with respect to CLK3 was obvious increased from 1.8 nM to more than 10 μM, suggesting that CLK1/4 may also be ideal targets of L41.^148^ The study further indicated that L41 displayed a neuroprotective role in glutamate-induced HT22 cell death.^148^ Moreover, Leucettine L41 decreased the phosphorylation of SRp75 in HMEC-1 cells. In addition, a CLK1 minigene transgenic model in Hela cells indicated that L41 treatment significantly elevated the percentage of exon inclusion of CLK1 itself.^150^

More compounds have been shown to inhibit CLKs activities and/or other CMGC kinases; however, detailed molecular mechanisms and in vivo experimental evidence are currently sparce. For example: Compound 3A5,^151^ ML315,^152^ SRI-29329,^153^ ML167,^154^ ML106,^155^ and BM07114^156^ still lack the pre-clinical data to support their anti-diseases efficiency. The constant efforts that uncover the underlying potential of CLKs inhibitors to prevent various diseases will pave the path to drug development and facilitate the progress of diseases treatment.

## Conclusions and future perspectives

Protein kinases, which phosphorylate specific substrate moieties, play critical roles in cell growth and differentiation. Substrate phosphorylation by kinases controls diverse cellular processes.^157^ Abnormal expression or dysfunction of protein kinases lead to many human diseases, including cancer. Many protein kinases are closely related to human diseases, for example, AURKA,^158^ AKT,^159^ CDK12,^160^ CDK15,^161^ LIMK,^162^ CLKs,^163^ AMPK,^164^ and p38 MAPK.^165^

CLKs participate in the phosphorylation of key proteins that regulate cellular metabolism and various signaling pathways.^133,166^ Irregular expression of CLKs and the dysregulation of alternative splicing have been identified in several human diseases; therefore, CLKs have emerged as a new class of disease hallmarks.^167–169^ Accumulating evidence has revealed the importance of CLKs in various physiological processes such as Duchenne muscular dystrophy, Alzheimer’s disease, and cancer. However, there are still limitations in the current understanding of CLKs. Firstly, the relationship between CLKs, immunotherapy, and the tumor microenvironment is currently unknown. Next, animal disease models, including CLKs conditional knockout or in situ disease models, are needed to fully understand the importance of CLKs in disease occurrence and development. Given the recent discovery showing that oxidation of the Met307 residue of CLK4 disrupted its kinase activity,^126^ more studies should be carried out to explore posttranslational modifications able to regulate CLKs function and expression. Moreover, the Q607R mutation within the CLK3 kinase domain enhanced c-Myc activity in promoting CCA,^124^ indicating the importance of CLKs somatic mutations in human diseases. Therefore, a mutation map of different CLKs detailing the functional consequences of specific mutations will contribute to explore the underlying molecular mechanism of CLKs. Therefore, continued investigation of CLKs functionality using high throughput sequencing and animal models are required to foster a more complete understanding of CLKs in human diseases occurrence and progression.

Due to the essential role of CLKs in human diseases, significant efforts have been made to study CLKs inhibitors using kinase screening technologies and pharmacological approaches. Most inhibitors have been well studied and have shown satisfactory anti-neurodegeneration, anti-inflammation, anti-viral, and anti-cancer effects by inhibiting the expression and activities of CLKs. However, there are still challenges associated with current CLKs inhibitors, and efforts can be made to achieve the following goals for disease therapeutics: 1) Targeted inhibition of specific CLKs for precision therapy. Most small molecular CLKs targeting drugs are broad-spectrum inhibitors, thus, specific inhibitors able to target CLK1, -2, -3, or -4 are needed to achieve greater therapeutic effect to reach a more precision therapy. 2) Development of pan-CLKs inhibitors to achieve heightened anti-cancer effects. The dysfunction and irregular expression of different CLKs members are concomitant in various human diseases, thus, the improvement of pan-CLKs inhibitors can help to maximize the treatment efficacy and benefit more patients in the clinic. 3) Enhance the specificity and selectivity of inhibitors to CLKs compared with other homologous CMGC family proteins through structural optimization. Many CLKs inhibitors show slight or strong off-target effects on other homologous kinases, such as, DYRKs and SRPKs. 4) Combination therapies of CLKs inhibitors with other compounds to achieve synergistic effects in the treatment of various diseases. Aiko et al. noted that treatment with CLK-IN-T3 in combination with the Bcl-xL/Bcl-2 inhibitor ABT-263 synergistically induced caspase 3/7-dependent apoptosis in A2780 and HCT116 cells compared to CLK-IN-T3 treatment alone.^132^ Meanwhile, E7107, an SF3b-targeting splicing modulator, synergistically enhanced apoptosis in NSCLC cell lines when combined with ABT-263.^170^ These findings indicate that the combination strategy of targeting CLKs with other inhibitors is an appropriate splicing molecular-based approach for future clinical development. It is noteworthy that certain CLKs inhibitors were recognized as ideal lead compounds for structural optimization to obtain more specific CLKs inhibitors. Moreover, a genetic function algorithm support vector regression (GFA-SVR)^171^ and ligand- or structure-based drug optimization^172,173^ provided predictive models and effective methods for screening and optimizing of the potential of CLKs inhibitors. Nonetheless, research on CLKs-based therapy is still in the initial stage; the cognition and development of CLKs inhibitors will undoubtedly progress upon more in-depth investigation.

In conclusion, CLKs facilitate biological processes through the phosphorylation or modulation of their downstream targets in a splicing or non-splicing-dependent manner. Changes in CLKs activity or expression level are closely related to pathological processes. CLKs inhibitors have shown significant therapeutic effects in various human diseases including neurodegenerative diseases, inflammatory diseases, viral replication, and cancer. To our knowledge, most of the compounds discussed in this review satisfy the criteria for chemical biology probes and have excellent prospects for the development of novel medicines. Given the essential role of CLKs in various human diseases, it can be expected that targeting CLK kinases may prove clinically beneficial in the future.
